# The role of alcohol control policy on the level of alcohol consumption in member states of the Association of Southeast Asian Nations 2000–2022: identifying trends and country clusters for further analyses

**DOI:** 10.7189/jogh.16.04150

**Published:** 2026-06-26

**Authors:** Jürgen Rehm, Daniela Correia, Gianna Gayle H Amul, Ian Yi Han Ang, Surasak Chaiyasong, Pheak Chhoun, Chean Lin Chong, Andrea Mong Rui Chua, Noran N Hairi, Enjeline Hanafi, Hoang Thi My Hanh, Ahmed S Hassan, Kyaw Ko Ko Htet, Norli Abdul Jabbar, Huan Jiang, Wah Yun Low, John Robert Carabeo Medina, Belinda J. Murtani, Jiraluck Nontarak, Sok King Ong, Pol Rovira, Kevin D Shield, Kristiana Siste, Vathsana Somphet, Vanphanom Sychareun, Chansathit Taikeophithoun, Yik-Ying Teo, Vassana Thammavongsa, Wen Ting Tong, Polathep Vichitkunakorn, Nguyen The Vinh, Wit Wichaidit, Andreas Suryo Wijaya, Qian Yang, Siyan Yi, Nurhaliza Zakariah, Ko Ko Zaw, Nyi Nyi Zayar, Hafsah Alwafa Zulakmal, Sawitri Assanangkornchai, Bundit Sornpaisarn

**Affiliations:** 1 Institute for Mental Health Policy Research, Centre for Addiction and Mental Health, Toronto, Ontario, Canada; 2 Campbell Family Mental Health Research Institute, Centre for Addiction and Mental Health, Toronto, Ontario, Canada; 3 PAHO/WHO Collaborating Centre at CAMH, Toronto, Ontario, Canada; 4 Dalla Lana School of Public Health, University of Toronto, Toronto, Ontario, Canada; 5 Department of Psychiatry, Faculty of Medicine, University of Toronto, Toronto, Ontario, Canada; 6 Faculty of Medicine, Institute of Medical Science, University of Toronto, Toronto, Ontario, Canada; 7 Center for Interdisciplinary Addiction Research, Department of Psychiatry and Psychotherapy, University Medical Center Hamburg-Eppendorf, Hamburg, Germany; 8 Program on Substance Abuse & WHO European Region Collaboration Centre, Public Health Agency of Catalonia, Barcelona, Catalonia, Spain; 9 WHO Regional Office for Europe, Copenhagen, Denmark; 10 EPIUnit – Instituto de Saúde Pública, Universidade do Porto, Porto, Portugal; 11 Laboratório para a Investigação Integrativa e Translacional em Saúde Populacional, Porto, Portugal; 12 FORUT, Gjøvik, Norway; 13 Ateneo School of Government, Quezon City, Philippines; 14 Saw Swee Hock School of Public Health, National University of Singapore and National University Health System, Singapore; 15 Social Pharmacy & Alcohol and Health Promotion Policy Research Units, Faculty of Pharmacy, Mahasarakham University, Thailand; 16 KHANA Centre for Population Health Research, Phnom Penh, Cambodia; 17 PAPRSB Institute of Health Sciences, Universiti Brunei Darussalam; 18 Department of Social and Preventive Medicine, Faculty of Medicine, Universiti Malaya, Kuala Lumpur, Malaysia; 19 Department of Psychiatry, Faculty of Medicine, Universitas Indonesia – dr. Cipto Mangunkusumo General Hospital, Jakarta, Indonesia; 20 Health Strategy and Policy Institute, Vietnam, Ministry of Health, Hanoi, Vietnam; 21 Centre for Alcohol Studies, Prince of Songkla University, Hat Yai, Songkhla, Thailand; 22 National Centre of Excellence for Mental Health, Selangor, Malaysia; 23 Department of Social and Preventive Medicine, Faculty of Medicine, Universiti Malaya, Kuala Lumpur, Malaysia; 24 Institute of Clinical Epidemiology, National Institutes of Health, University of the Philippines Manila, Manila, Metro Manila, Philippines; 25 Department of Epidemiology, Faculty of Public Health, Mahidol University, Bangkok, Thailand; 26 Department of Epidemiology and Biostatistics, Schulich School of Medicine and Dentistry, Western University, London, Ontario, Canada; 27 Faculty of Public Health, University of Health Sciences, Lao PDR; 28 Department of Primary Care Medicine, Faculty of Medicine, Universiti Malaya, Kuala Lumpur, Malaysia; 29 Health Policy Research Center, Faculty of Medicine, Prince of Songkla University, Hat Yai, Songkhla, Thailand; 30 Department of Family and Preventive Medicine, Faculty of Medicine, Prince of Songkla University, Hat Yai, Songkhla, Thailand; 31 Department of Epidemiology, Faculty of Medicine, Prince of Songkla University, Hat Yai, Songkhla, Thailand; 32 Disease Control Division, Ministry of Health Malaysia, Malaysia; 33 Faculty of Health Science, STI Myanmar University, Yangon, Myanmar

## Abstract

**Background:**

In his historical studies of current high-income countries, Angus Deaton demonstrated a correlation between a country’s prosperity and the health of its population. His conceptual framework has recently been expanded to include the role of alcohol. In this ecological study, we test the key assumptions of the expanded framework and classify countries with respect to both their levels of alcohol consumption and alcohol control policies to economic development.

**Methods:**

We explored linear trends from 2000 to 2022 in gross domestic product *per capita* at purchasing power parity (GDP-PPP *per capita*), life expectancy, and adult alcohol *per capita* consumption (APC) in 10 member states of the Association of Southeast Asian Nations (ASEAN), grouping them through cluster analyses based on these three variables. We also scored and ranked the countries based on their alcohol control policies. Lastly, we used generalised least squares models, accounting for temporal autocorrelation, to evaluate the associations between GDP-PPP *per capita*, life expectancy, and APC.

**Results:**

We corroborated Deaton’s conclusions that increases in economic wealth were associated with increases in life expectancy, with the largest improvements seen in low-income countries and the smallest in high-income countries. Economic transition was consistently related to increases in the level of alcohol consumption in low-income economies, with the exception of Muslim-majority countries. Following their transition to a lower middle-income status, some ASEAN countries appear to have implemented more effective alcohol control policies that halted further increases in their levels of consumption. In Muslim-majority countries, the level of consumption was low, irrespective of the level of economic development.

**Conclusions:**

The implementation of alcohol control policies prevents further increases in alcohol consumption and attributable harm and thus allows countries undergoing economic transition to reap the full health benefits of economic development.

In his seminal work ‘The great escape: health, wealth, and the origins of inequality’, Angus Deaton demonstrated how economic development in a country moving from poverty to a high-income economy positively impacted health and life expectancy [1]. Countries are classified in various income categories in a standardized manner by the World Bank [2]. Deaton’s framework was recently expanded to include the role of alcohol consumption, alcohol control policies, and related factors such as culture or religion [3]. This study attempts to empirically examine some of these processes in the Association of Southeast Asian Nations (ASEAN) region (**Figure 1**) [4].

**Figure 1:**
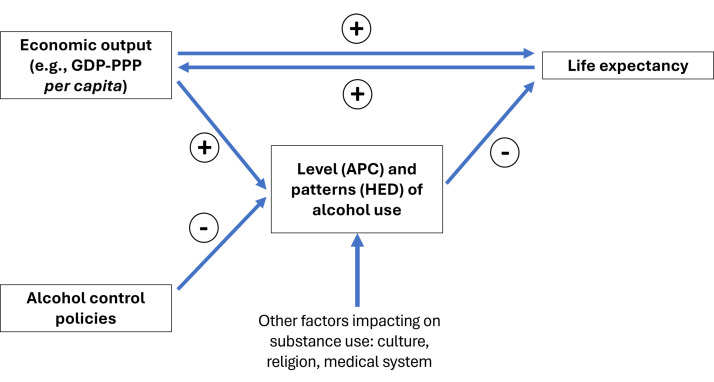
Economic development, alcohol consumption, alcohol control policies, and life expectancy: a conceptual model. Adapted from Rehm *et al*. (published under CC BY-NC-ND 4.0 license), with permission [3]. APC – adult alcohol *per capita* consumption, GDP-PPP – gross domestic product at purchasing power parity, HED – heavy episodic drinking.

The main independent variable in the model is economic output, which impacts on both the level of alcohol consumption and life expectancy. The former has largely been attributed to increases in purchasing power and the alcohol industry’s intense marketing efforts in low- and middle-income countries with high rates of economic growth [5], while latter results from improved overall living conditions and a reduction in disease and perinatal and infectious causes of death [6]. Finally, alcohol use impacts negatively on life expectancy as a consequence of alcohol-attributable mortality [7,8], which in turn negatively affects economic output [9]. However, the level of alcohol use can be reduced and the patterns (*e.g.* frequency and temporal distribution of consumption) improved through the implementation of effective alcohol control policies [10,11], and/or by cultural or religious factors (most importantly in Muslim-majority countries [12]).

A first empirical test of the model using all countries in the Western Pacific Region of the World Health Organization (WHO) that transitioned between low- and lower middle-income status between 2000 and 2020 corroborated the main relationships between economic development, level of alcohol consumption, and life expectancy [13], without including alcohol control policies or religion in the model. We will, therefore, include these constructs in this modelling study of ASEAN countries. These ASEAN member states were selected as they have experienced substantial economic growth since 2000, which was associated, as predicted by Deaton [1], with substantial health gains: their economies, on average – as measured by the gross domestic product *per capita* at purchasing power parity (GDP-PPP; for definition, see Glossary in the **Online Supplementary Document**) – grew at an annual rate of almost 6% [14], coupled with an annual increase in life expectancy of 0.3% [15]. This means that, on average, their GDP-PPP *per capita* increased by 18 818 international dollars between 2000 and 2022.

However, it is unclear if the relationship between economic development and life expectancy holds for all countries or if it is influenced by other factors, such as level of alcohol use, alcohol control policies, or religion influence. To explore this question based on the above-specified framework, we attempted to establish an empirically based classification system of countries in the ASEAN region. We hope this system will not only guide future research on the role of alcohol use in different environments, but also assist in the monitoring of the burden attributable to alcohol consumption and the implementation and evaluation of alcohol control policies.

## METHODS

We retrieved comparable data for all ASEAN countries for the 2000–2022 period from international organisations such as the World Bank (GDP-PPP *per capita*, life expectancy), the WHO (adult alcohol *per capita* consumption (APC); tax rates) and the World Population Review (Appendix S1 in the **Online Supplementary Document**). Local experts separately retrieved data on alcohol policies from each ASEAN country [16].

### Policy analysis

For all countries, we identified the timing of the enactment of the SAFER policies outside of the healthcare system (increases in alcohol excise taxation, availability, and marketing restrictions, drink-driving control) with respect to the economic transition (*i.e.* before or after the first move in income classification, as per the World Bank, between 2000 and 2022). Similar to recent work on alcohol in the Baltics [17], we then constructed a scale of the potential impact of these policies based on various dimensions (Table S1 and Appendix S2 in the **Online Supplementary Document**). The scale was restricted to the three ‘best buy’ policies, which were initially recommended by the WHO to cost-effectively reduce the burden of non-communicable disease [18,19], but have been shown to have a wider impact on all-cause mortality and the burden of disease [20,21].

### Analysis of religion

Islam is the only major religion that effectively controls alcohol consumption through cultural stigma and legal measures, including outright bans on alcohol consumption [16]. Accordingly, we assigned a religious score of 0.05 for each 1% of the Muslim population within each ASEAN country, which we then combined with the policy score. Specific measures, including Brunei Darussalam’s ban on alcohol sales, are detailed under policy measures in Appendix S2 in the **Online Supplementary Document**.

### Cluster analysis based on trajectories

To explore similarities in temporal trends across countries, we analysed the trajectories of three key variables: APC, GDP-PPP *per capita*, and life expectancy, from 2000 to 2022 for the 10 ASEAN countries. For each country and variable, we estimated linear trends over time using ordinary least squares regression, through which we extracted three intercepts and three slope coefficients to characterise each trajectory for each country. We then applied hierarchical clustering to the 10 countries using these six coefficients. Euclidean distance was used as the dissimilarity metric, and Ward’s minimum variance method was applied for linkage. We selected this approach over alternative clustering methods because it does not require pre-specification of the number of clusters and allows for easy visual inspection of the resulting dendrogram to guide cluster selection. We determined the number of clusters based on visual inspection of the dendrogram and the elbow method [22].

### Other statistical analysis

We fitted generalised least squares (GLS) models to the time series of the three indicators to assess the associations between economic development and life expectancy and APC. This method accounted for autocorrelation in the longitudinal data, which was modelled using a compound symmetry correlation structure. Model adequacy was assessed by inspecting autocorrelation function plots of the normalised residuals.

We estimated two main model specifications: one with APC and another with life expectancy specified as the dependent variable. In both, GDP-PPP was the independent variable, divided by 1000 to improve interpretability of the regression coefficients. We fitted these models separately for different subsets of countries and time periods, including, but not limited to, all 10 ASEAN countries and by clusters. For each fitted model, we evaluated the assumption of normality of residuals using histogram plots. When deviations from normality were observed, the dependent variable was log-transformed prior to refitting the GLS model. We used R, version 4.3.3 (R Core Team, Vienna, Austria) for all statistical analyses.

## RESULTS

### The first classification of countries

The hierarchical clustering grouped the 10 countries into two main clusters: the first included Brunei Darussalam, Indonesia, Malaysia, Philippines, Singapore, and Thailand, while the second included Cambodia, Lao PDR, Myanmar, and Viet Nam. Countries in the first group were characterised by relatively stable levels of APC, higher GDP-PPP, and higher life expectancy. In contrast, countries in the second group generally showed increasing trends in alcohol consumption alongside lower but increasing levels of GDP-PPP and life expectancy (Appendix S3 in the **Online Supplementary Document**). While the dendrogram suggested two broad clusters, the elbow method indicated that five clusters would be optimal. This solution would retain the two initial clusters, while identifying Viet Nam, Singapore, and Thailand as distinct, single-country clusters, reflecting their unique trajectories for some of the indicators being tracked. However, opting for this solution would lead to losing the ability to compare countries within clusters for the three clusters containing only one country. Following the identification of two main clusters, we separated the Muslim-majority countries from the first category due to their consistently low APC levels (Appendix S3 in the **Online Supplementary Document**). We thereby arrived at three groups of countries for further analyses (Appendix S4 in the **Online Supplementary Document**):

− cluster 1 (Muslim-majority countries): Brunei Darussalam, Indonesia, and Malaysia. These countries maintained an APC lower than 1 L pure alcohol throughout the entire time period examined.

− cluster 2 (non-Muslim-majority countries): the Philippines, Singapore, and Thailand. These nations are characterised by relatively stable APCs and high income levels.

− cluster 3 (non-Muslim-majority countries): Cambodia, Lao PDR, Myanmar, and Viet Nam. These countries showed increasing APCs and held a low-income classification in 2000.

An overview of country characteristics by these clusters can be found in **Table 1**.

**Table 1:** Religion, economic characteristics, and alcohol consumption of 10 ASEAN countries

	Cluster 1: Muslim-majority countries			Cluster 2: countries starting at lower middle-income or higher in 2000			Cluster 3: countries starting at the level of low-income in 2000			
	**BRN**	**IDN**	**MYS**	**PHL**	**SGP**	**THA**	**KHM**	**LAO**	**MMR**	**VNM**
**Cultural factor**										
Proportion of Muslim population in %	78.8	88.3	61.3	11.0	15.6	5.2	2.0	0.0	4.3	0.1
**Economic factor**										
GDP-PPP per capita in 2000 in 1000 international dollars	73.09	4.64	13.03	3.37	43.78	7.29	1.26	1.79	0.95	2.61
GDP-PPP per capita in 2022 in 1000 international dollars	81.80	14.29	34.37	10.13	141.91	22.22	6.92	8.77	5.73	13.85
Relative increase in GDP-PPP in %	11.9	207.9	163.8	201.0	224.1	204.8	450.9	388.7	504.2	429.9
Economic status in 2000	HIC	LIC	UMIC	LMIC	HIC	LMIC	LIC	LIC	LIC	LIC
Economic status in 2022	HIC	UMIC	UMIC	LMIC	HIC	UMIC	LMIC	LMIC	LMIC	LMIC
Number of economic status transitions and the year(s) of occurrence	0	2 (2002, 2019)	0	0	0	1 (2010)	1 (2015)	1 (2010)	1 (2014)	1 (2009)
**Alcohol consumption**										
APC in 2000	0.34	0.09	0.71	5.85	2.12	8.30	0.80	8.63	0.26	3.88
APC in 2022	0.71	0.10	0.93	6.01	2.00	7.86	6.37	11.48	1.87	11.54
APC change in 2022–2000	+0.37	+0.01	+0.22	+0.16	−0.12	−0.46	+5.57	+2.85	+1.61	+7.66

APC – alcohol *per capita* consumption, BRN – Brunei Darussalam, GDP-PPP – gross domestic product *per capita* at purchasing power parity, HIC – high-income country, IDN – Indonesia, KHM – Cambodia, LAO – Lao PDR, LIC – low-income country – LMIC – lower middle-income country, MMR – Myanmar, MYS – Malaysia, PHL – Philippines, SGP – Singapore, THA – Thailand, UMIC – upper middle-income country, VNM – Viet Nam

### The association between economic development and life expectancy

There was a consistent positive association between GDP-PPP and life expectancy at birth (Appendix S5 in the **Online Supplementary Document**). However, the association was stronger during periods when the countries were at lower income levels. Few differences in the strength of the association between the three clusters of countries were evident.

These descriptive results were corroborated by the regression analyses (**Table 2**). Specifically, an increase of international USD 1000 was associated with a 2.8-year gain in life expectancy for the low-income group, compared to only 0.05 years for the high-income group (average increase of about 0.13 years).

**Table 2:** Association of GDP-PPP *per capita* (in 1000 international dollars) with life expectancy and APC in ASEAN countries from 2000–2022, during periods of different income levels and by cluster

	Coefficient (95% CI)	*t*-value	*P*-value
**Life expectancy and GDP-PPP**			
Ten ASEAN countries	0.134 (0.115, 0.153)	13.838	<0.001
During LIC period	2.835 (2.198, 3.472)	8.724	<0.001
During LMIC period	0.524 (0.324, 0.723)	5.135	<0.001
During UMIC period	0.096 (−0.034, 0.226)	1.451	0.155
During HIC period†	0.0007 (0.0005, 0.0010)	5.7122	<0.001
Cluster 1†	0.0009 (0.0006, 0.0012)	6.0372	<0.001
Cluster 2†	0.0015 (0.0013, 0.0017)	14.2406	<0.001
Cluster 3†	0.0170 (0.0146, 0.0195)	13.5976	<0.001
**APC and GDP-PPP**			
10 ASEAN countries†	−0.011 (−0.018, −0.005)	−3.465	<0.001
During LIC period†	0.127 (−0.199, 0.452)	0.762	0.450
During LMIC period	0.036 (−0.150, 0.221)	0.376	0.708
During UMIC period†	−0.029 (−0.102, 0.043)	−0.791	0.434
During HIC period	0.005 (0.0003, 0.0099)	2.099	0.042
Cluster 1	0.004 (0.002, 0.007)	3.142	0.002
Cluster 2	−0.021 (−0.029, −0.014)	−5.556	<0.001
Cluster 3†	0.189 (0.159, 0.218)	12.519	<0.001

APC – alcohol *per capita* consumption, ASEAN – Association of Southeast Asian Nations, CI – confidence interval, GDP-PPP – gross domestic product *per capita* at purchasing power parity, HIC – high-income countries, LIC – lower income country, LMIC – lower middle-income country, UMIC – upper-middle income country

*Cluster 1: Brunei Darussalam, Indonesia, and Malaysia. Cluster 2: Philippines, Singapore, and Thailand. Cluster 3: Cambodia, Lao PDR, Myanmar, and Viet Nam.

†The outcome (life expectancy or APC) was log-transformed.

### The association between economic development and APC

We found no clear and consistent associations between economic development and APC. While there was an overall inverse relationship for all ASEAN countries, the countries in cluster 3, which started in the year 2000 in the low-income category, showed increases of 19% in APC associated with increases of international USD 1000 in GDP-PPP *per capita* (**Table 2**; Appendix S5 in the **Online Supplementary Document**).

### The association between alcohol control policies and alcohol consumption levels

Overall, a clear cluster-specific pattern of policy enactments emerges from our analysis of alcohol control policies and the related scoring and ranking based on scales reflecting enacted policies and religious factors (**Table 3**; Tables S1–3 in the **Online Supplementary Document**). Among Muslim-majority countries (cluster 1), Indonesia and Malaysia rely primarily on the informal control inherent in religious beliefs, whereas Brunei Darussalam has implemented a ban on alcohol sales. Consequently, Brunei Darussalam ranks first among 10 ASEAN nations, while Malaysia and Indonesia rank seventh and ninth, respectively. However, when combining policy scores with religious factors (percentage of Muslim population), these three countries rank as the top three for alcohol control, as overall the level of APC remained under 1 L, considerably below the global [23] or regional averages.

**Table 3:** Scores and ranking of alcohol control policies, religious factors, and APC changes for 10 ASEAN countries in 2000–2022

Policy score (score criteria)	Code*	BRN	IDN	MYS	PHL	SGP	THA	KHM	LAO	MMR	VNM
Alcohol control policy											
Tax score		17	8	10	17	14	14	9	10	10	6
Standardised taxation score (0–5)	A	5.00	2.35	2.94	5.00	4.12	4.12	2.65	2.94	2.94	1.76
Availability control		12	2	4	1	8	10	0	5	6	4
Standardised availability control score (0–5)	B	5.00	0.83	1.67	0.42	3.33	4.17	0.00	2.08	2.50	1.67
Advertising control score		8	3	3	1	1	6	0	3	3	4
Standardised advertising control score (0–2.5)	C	2.50	0.94	0.94	0.31	0.31	1.88	0.00	0.94	0.94	1.25
Average standardised policy score (0-5)		5.00	1.65	2.22	2.29	3.11	4.06	1.06	2.38	2.55	1.87
Ranking policy scores	D	1	9	7	6	3	2	10	5	4	8
Religious factors											
Percentage Muslim (%)		78.8	88.3	61.3	11.0	15.6	5.2	2.0	0.0	4.3	0.1
Additional religious score (1% = 0.05 score)		3.94	4.42	3.07	0.55	0.78	0.25	0.10	0.00	0.20	0.01
Sum of policy score and religious score		8.94	6.07	5.29	2.84	3.89	4.31	1.16	2.38	2.75	1.88
Standardised policy score and religious score (0**–**5)	E	5.00	3.39	2.96	1.59	2.17	2.41	0.65	1.33	1.54	1.05
Ranking of policy and religious scores		1	2	3	6	5	4	10	8	7	9
APC absolute change from 2000 to 2022†		+0.37	+0.01	+0.22	+1.16	−0.12	−0.46	+5.57	+2.85	+1.61	+7.66
APC in 2022		0.71	0.10	0.93	6.01	2.00	7.86	6.37	11.48	1.87	11.54
Ranking of APC changes		5	3	4	6	2	1	9	8	7	10

APC – alcohol *per capita* consumption, ASEAN – Association of Southeast Asian Nations, BRN – Brunei Darussalam, IDN – Indonesia, KHM – Cambodia, LAO – Lao PDR, MMR – Myanmar, MYS – Malaysia, PHL – Philippines, SGP – Singapore, THA – Thailand, VNM – Viet Nam

*Code drawn from Table S1 in the **Online Supplementary Document**.

†Data drawn from **Table 1**.

As for cluster 3 – comprising countries with low-income levels in 2000 (Cambodia, Lao PDR, Myanmar, and Viet Nam) – only relatively weak policies were implemented relatively late in the transition, and no cultural or other factors seem to have impacted the rising APC trends until 2019. In 2019 and 2020, consumption temporarily decreased due to new policy implementations and the COVID-19 pandemic, respectively. Cluster 2, which included countries that were lower middle-income or higher in 2000 (the Philippines, Singapore, Thailand), seem to have implemented more effective alcohol control policies earlier and consequently experienced fairly stable consumption levels. Among the 10 ASEAN countries, cluster 3 countries ranked seventh to tenth based on the combined scores of the policy and religious factors, while cluster 2 countries occupied the fourth, fifth, and sixth ranks.

**Figure 2:**
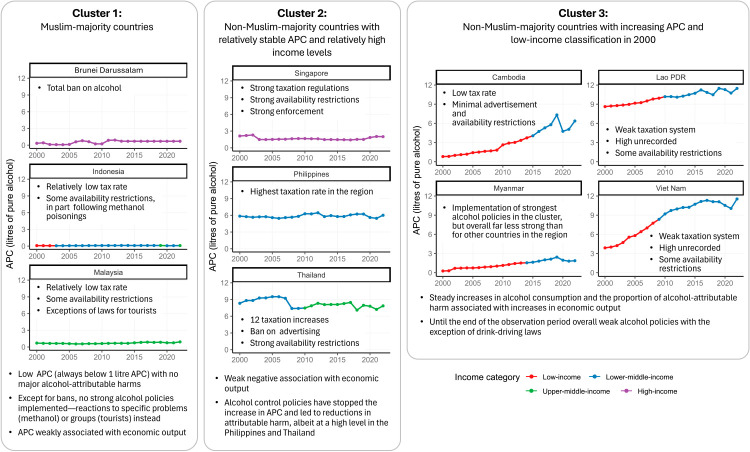
Summary results for the three country clusters. APC – adult alcohol *per capita* consumption.

Overall, when religion and policy factors are taken together, the countries align perfectly in the clusters. Cluster 1 can be characterised by the overall lowest level of consumption, even though some relative increases were seen (ranked first to third overall). Cluster 2 is characterised by overall successful efforts to halt further increases of APC, albeit at different levels, with Thailand ranking significantly above the global average, the Philippines slightly above, and Singapore clearly below (countries ranked fourth to sixth overall). Finally, cluster 3 experienced increases due to not implementing a sufficiently strong set of policies to stop the increase in alcohol consumption (ranked seventh to tenth overall). It should be noted that within cluster 3, Myanmar, the country with the relatively strongest policies, clearly had a slower trajectory in increasing alcohol consumption (**Figure 2**, **Table 3**).

## DISCUSSION

While we found overall confirmation for the conceptual model on the impact of economic development on health and alcohol use, we summarise current knowledge based on the experience of ASEAN countries as follows. Economic development was associated with increases in life expectancy, with the main effects occurring during the phase of lower income. This means that the higher the economic development, the lower the average gain in life expectancy per unit increase of GDP-PPP *per capita*. With respect to the association with APC, as postulated, alcohol control policies and culture or religion – in the case of ASEAN member states, Islam – were associated with slowing or halting the impact of economic development on alcohol consumption. Without either of these factors, there seem to be uncontrolled increases in alcohol consumption, which in the case of ASEAN member states were only stopped during the COVID-19 pandemic. As the level of alcohol consumption is one of the main contributors to alcohol-attributable mortality [24], uninterrupted and uncontrolled increases in APC will likely lead to decreases in life expectancy [13].

Before discussing the implications of these findings, we must acknowledge the limitations of our approach. First, we have examined trends in ecological data to set up a classification for the subsequent analyses. However, we cannot establish causality based on the current analyses alone; further analyses with a higher degree of statistical control are necessary to rule out potential alternative explanations [21,25]. Second, we have not yet established a final methodology to estimate the impact of APC on life expectancy, even though alcohol consumption is associated with lower life expectancy through premature alcohol-attributable deaths [23,26]. To establish a final methodology, we would need to employ life expectancy tables and incorporate some assumptions regarding competing causes of deaths, as suggested elsewhere [13]. Third, due to the fact that, with only one exception [27], there are no established Asian scales for alcohol control policies to score countries, we constructed an *ad hoc* scale based on the WHO’s best buys, which appear in all alcohol control policy scores, albeit with different scales or rules for scoring them (Appendix S2 in the **Online Supplementary Document**). In contrast to the approach used with some scales [28], marketing bans in this study have been scored using half the points allotted to the other two best buys. While there is some evidence for an impact on consumption as a result of marketing restrictions [29,30], a recent systematic review concluded that there is not enough evidence for this type of policy to be considered a best buy [31–33]. Classifying these measures as ‘best buys’ thus remains controversial due to varying effectiveness in reducing consumption [32,34] and the timing of their impact [17,35,36]. Nevertheless, the same classification into clusters would have resulted from us focusing strictly on taxation and availability restrictions alone – the two best buys that are not at all controversial [10,21,37]. Data limitations also persist, particularly in terms of the absence of comparable indicators for policy enforcement. For instance, Myanmar has a 1917 law that serves as a prime example regulating the manufacturing, possession, sale, import, export, and transport of alcohol. There are some doubts as to what degree this law has been enforced in this country, but its current political situation (*i.e,* country undergoing a civil war marked by economic deterioration, mass displacement, and a severe humanitarian emergency) does not allow for further inquiry. However, even in ASEAN countries without civil conflict, enforcement data for major policies remain spotty and contradictory. However, the extent of unrecorded consumption [38,39], which is collected in a standardised way *via* the WHO STEPS survey in the region, is available to us [40]. Specifically for Brunei Darussalam, it should be noted that the WHO data on APC was unstable due to its small population size, and the rough methodology based on food balance sheets employed by the Food and Agriculture Organization of the United Nations that provided data estimated up until 2013 [41,42]. The data for the years following 2013 were imputed, which is why the results for this country may be biased.

Despite these limitations, the results supporting this classification method underscore the key roles of alcohol control policy and religion during a country’s economic transition (*i.e.* we found no other strong cultural factors within ASEAN countries). This result can additionally be corroborated by recent analyses for India [43] and China [44]. In both cases, policies were found to be responsible for halting further increases in APC. In India, this was achieved through the classic best buys of taxation and availability restrictions, including state-level bans [43]; in China, the primary driver was the anti-corruption campaign, though it was not originally intended to target APC [44]. Due to a lack of effective alcohol control policies and the presence of cultural barriers, alcohol consumption seems to have increased steadily to high overall levels. Consequently, Viet Nam and Lao PDR now rank within the top 20 countries for average global consumption [45], exhibiting the expected associated negative consequences in terms of attributable burden of disease and social harm.

Future research on the role of alcohol use in ASEAN countries should be sensitive to the potential impacts of religion and any new alcohol control policies implemented. The enforcement level of these policies should be incorporated not only nationally but also into all global monitoring systems to enable accurate comparisons to be made between countries. Moreover, countries should ideally be compared to countries placed within the same cluster, such as through interrupted time series analyses [21].

## CONCLUSIONS

Strong alcohol control policies should be implemented to prevent increases in consumption and attributable harm in countries without cultural or religious barriers to alcohol consumption, so that they can reap the full health benefits of economic development. Without such measures, increases in APC will lead to a decrease in life expectancy, thus slowing down economic development.

## Additional material


Online Supplementary Document
