# Supplementary material for: The role of alcohol control policy on the level of alcohol consumption in member states of the Association of Southeast Asian Nations 2000–2022: identifying trends and country clusters for further analyses
Source: J Glob Health. 2026 Jun 26;16:04150. doi: 10.7189/jogh.16.04150 (PMC13307440; doi:10.7189/jogh.16.04150)
Supplement: Online Supplementary Document [file jogh-16-04150-s001.pdf]

Supplement to: Rehm J, Correia D, Amul GGH, Ang IYH, Chaiyasong S, Chhoun P, Chong CL, Chua AMR, Hairi NN, Hanafi E, Hanh HT, Hassan AS, Htet KKK, Jabbar NA, Jiang H, Low WY, Medina JRC, Murtani BJ, Nontarak J, Ong SK, Rovira P, Shield KD, Siste K, Somphet V, Sychareun V, Taikeophithoun C, Teo YY, Thammavongsa V, Tong WT, Vichitkunakorn P, Vinh NT, Wichaidit W, Wijaya AS, Yang Q, Yi S, Zakariah N, Zaw KK, Zayar NN, Zulakmal HA, Assanangkornchai S, Sornpaisarn B. The role of alcohol control policy on the level of alcohol consumption in member states of the Association of Southeast Asian Nations 2000–2022: identifying trends and country clusters for further analyses. J Glob Health. 2026;16:04150.

Appendices

The role of alcohol control policy on the level of alcohol consumption in Member States of the Association of Southeast Asian Nations (ASEAN) 2000–2022: identifying trends and country clusters for further analyses

Table of Contents

Appendices 1

The role of alcohol control policy on the level of alcohol consumption in Member States of the Association of Southeast Asian Nations (ASEAN) 2000–2022: identifying trends and country clusters for further analyses 1

Glossary 4

Appendix S1: Data sources 5

Appendix S2: Scoring of alcohol control policies 6

Table S1: Criteria for alcohol control policy scoring...8

Table S2: Alcohol control policies in 10 ASEAN countries in 2000–2022 11

Table S3. Alcohol control policy enactments and timeliness among 10 ASEAN countries during 2000–2022 21

Table S4: Scores and ranking of alcohol control policies, religion factors, and APC for 10 ASEAN countries in 2000 24

Appendix S2 25

Figure S1: Overview of wealth in GDP-PPP *per capita* (in $ Int.), adult alcohol *per capita* consumption (APC), and life expectancy at birth in all 10 ASEAN Member States...25

Figure S2: Overview of wealth in GDP-PPP *per capita* (in $ Int.), adult alcohol *per capita* consumption (APC), and life expectancy at birth for the ASEAN region and by cluster, weighted by population size 26

Appendix S3: Cluster analysis based on trajectories 28

Figure S3: Estimated trajectories of APC, GDP-PPP, and life expectancy from 2000 to 2022 for each of the ten countries. Solid lines represent the linear regression fit used to derive intercept and slope coefficients for the clustering analysis 28

Figure S4: Dendrogram from hierarchical clustering based on six trajectory coefficients per country. 29

Figure S5: Elbow method plot for identifying the optimal number of clusters. 29

Figure S6: Countries grouped by the two-cluster solution, showing the trajectories of APC, GDP-PPP, and life expectancy from 2000 to 2022. Each line represents a country, colored by cluster assignment. 30

Figure S7: Countries grouped by the five-cluster solution, showing the trajectories of APC, GDP-PPP, and life expectancy from 2000 to 2022. Each line represents a country, colored by cluster assignment 31

Appendix S4: Association between GDP-PPP *per capita* and life expectancy and population alcohol consumption (APC) 32

Pearson correlations 32

Table S4: Pearson correlations between GDP-PPP *per capita* and life expectancy at birth, and APC 2000–2022 32

Appendix S5: Results of Generalized Least Squares (GLS) models evaluating the association of Gross Domestic Product *per capita* at purchasing power parity (GDP-PPP *per capita*) with Life Expectancy and with alcohol *per capita* consumption (APC) 33

Life Expectancy and GDP-PPP *per capita* 33

Grouping by clusters 38
APC with GDP-PPP *per capita* 41
Grouping by clusters 46
References 49

Glossary

Term	Definition
Adult alcohol *per capita* consumption (APC)	Total alcohol (sum of recorded and unrecorded adjusted for tourist consumption) consumed per adult (15+ years) in a calendar year, estimated in litres of pure alcohol (ethanol) **(1)**.
Association of Southeast Asian Nations (ASEAN)	An international organization that aims to promote economic growth, social progress, cultural development, peace, and stability in Southeast Asia. Its Member States comprise Brunei Darussalam, Cambodia, Indonesia, Lao PDR, Malaysia, Myanmar, the Philippines, Singapore, Thailand, and Viet Nam **(2)**.
Best buy policies	Evidence-based, cost-effective strategies recommended by the World Health Organization to reduce alcohol-attributable non-communicable diseases and mortality. These include increasing alcohol excise taxes, banning or restricting alcohol advertising, and reducing availability of alcoholic beverages (see text for references and more detail).
Economic transition	Shift of a country from one income level to another as classified by the World Bank. The income categories include low-income, lower middle-income, upper middle-income, and high-income. Country classifications are updated each year on July 1 based on the gross national income *per capita* of the prior year **(3)**.
Gross Domestic Product *per capita* at purchasing power parity (GDP-PPP *per capita*)	GDP-PPP represents the economic output of goods and services per person in a country, expressed in international dollars using purchasing power parity rates to make prices comparable across countries. For comparability reasons, this indicator is standardized for population size **(4)**.
High-income country (HIC)	High-income countries, as defined by the World Bank, are those that have a gross national income *per capita* of greater than $14,005 (2023 threshold). For all income classifications, see **Economic transition.**
Current International dollar (Int$)	A hypothetical currency unit with equivalent purchasing power parity to a United States dollar USD at a given point in time.
Life expectancy	Life expectancy at birth is the average number of years that a newborn is expected to live, given the sex- and age-specific death rates in a particular year and jurisdiction (e.g., country, region).
Lower middle-income country (L-MIC)	Lower middle-income countries, as defined by the World Bank, are those that have a gross national income *per capita* of $1,146 and $4,515 (range for the year 2023). For all income classifications, see **Economic transition.**
Low-income country (LIC)	Low-income countries, as defined by World Bank, are those that have a gross national income *per capita* of $1,145 or less (range for the year 2023). For all income classifications, see **Economic transition.**
SAFER interventions	Five cost-effective interventions aimed at reducing alcohol-related harm, including the three "**best buys"** (alcohol excise taxation increases to raise prices, alcohol availability and marketing restrictions), plus drink-driving countermeasures, access to screening, brief interventions and treatment **(5)**.
Upper middle-income country (U-MIC)	Upper middle-income countries, as defined by the World Bank, are those that have a gross national income *per capita* of $4,516 and $14,005 (2023 range). For all income classifications, see **Economic transition.**

Appendix S1: Data sources

For all ASEAN countries, we collected data from the World Bank between 2000 and 2022 on GDP-PPP *per capita* **(6)** and life expectancy **(7)** and from the WHO's World Health Statistics on adult alcohol *per capita* consumption (APC) **(8)**. The WHO calculates APC as the sum of all recorded, unrecorded, and tourist consumption in a country (for an overview, see **(9)**). Recorded alcohol consumption data are often taken from taxation data, or from production data adjusted for imports or exports **(10)**. Unrecorded alcohol, i.e. alcohol not officially registered, is usually modelled based on surveys **(11, 12)**, usually from the WHO STEPS survey **(13)**. Tourist consumption is estimated based on UN data **(14)**. Data on the percentage of Muslims in the population in each country were drawn from the World Population Review **(15)**.

Data on alcohol policies were collected from each participant country of the ASEAN project by local experts **(16)** using the WHO SAFER policies' framework **(17)**. Participants also included any other policies considered to have a potential impact on consumption and harm. Data on percentages of alcohol excise tax to the retail price (% tax-to-price) were from the *Global Report on the Use of Alcohol Taxes* by the WHO **(18)**.

Appendix S2: Scoring of alcohol control policies

To compare alcohol control policies between countries, two steps are essential: first, all relevant policies need to be assessed, and then must be weighted to form an overall policy score (scoring). These procedures also underlie the ASEAN project. In a first step, we assessed all major alcohol control policies between the years 2000 and 2022 (see main text).

For scoring, we first researched the currently available existing alcohol control policy scoring tools **(19-25)**. These studies focussed on different geographical regions and domains. Brand et al. **(19)**, Madureira-Lima & Galea **(21)**, and Caswell et al. **(20)** were global in nature, focussing on between five and 10 policy domains, with 16 to more than 100 policy items assessed. The other attempts were more regional; Månsson and colleagues used seven domains for policies in the European Union **(25)**; Naimi and colleagues (2014) assessed 29 policies in six policy domains in Canada **(23)**; PAHO focused on 34 summary indicators in 10 policy areas for the WHO Americas Region **(24)**; and Naimi et al. addressed 84 policy items for the US. Finally, Amul and Etter **(26)** compared two ASEAN countries.

The major policy domains are also reflected in WHO documents such as the 10 alcohol policy areas recommended in the Global Strategy to Reduce the Harmful Use of Alcohol **(27)**, as well as the five SAFER interventions, including the three best buys **(5)**. These measures have received some empirical support **(28, 29)**.

As for the weighting of the domains, different mechanisms were used to comparatively scale alcohol control policies: equal weight, weighted based on effectiveness of the policies, weighted based on the evidence strength of the policies, or weighted by complex statistical methods. Several studies determined the weighting after performing a sensitivity analysis. Even though some of them claimed that their scoring was based on evidence of the policy effectiveness, they assigned different weights for the same policy. For example, Caswell et al. **(20)** and Brand et al. **(19)** gave the alcohol taxation the highest weight compared to other policies (four scoring points and three stars, respectively). However, Casswell gave three scoring points **(20)** while Brand gave only one star for marketing control policies **(19)**. Some studies separated scores for the policies in place and their implementation into two sub-scorings before eventually combining them again into a single score **(20, 23)**. For example, Naimi et al. **(23)** assigned a score of 3.225 if a U.S. State had a policy restricting hours of alcohol sales, and the additional implementation score of 0.5 if that policy allowed less than 16 hours of sales, but 0.2 if it allowed more than 16 hours of sales, etc. Other authors combined them into a single score at the outset; for example, **(24)** gave a score of four for a policy of comprehensive restrictions on either days or hours of sale for both on- and off-premise locations, three for comprehensive restrictions on either days or hours of sale for either on- or off-premise locations, two for partial restrictions on either days or hours of sale for both on- and off-premise locations, one for partial restrictions either days or hours of sale for either on- or off-premise locations, and zero for no restrictions at all **(24)**.

We applied a two-step analysis of the collected alcohol control policies of ASEAN countries: policy summarizing as well as scoring and ranking. For the first step, we summarized data on the country's alcohol control policies (actually implemented) based on four WHO SAFER **(5)** policies: taxation, availability control, advertising, and drink-driving. As indicated in the main text, we concentrated our scoring on the best buys **(29, 30)** due to their potentially higher impact (see details of scoring below). In addition, screening, brief intervention, and treatment policies were included in the healthcare system path of our conceptual diagram (see main text).

For the second step, three issues needed to be clear. (A) Since we were only interested in alcohol control policies that significantly affected alcohol consumption, we analyzed the three WHO best buy policies **(30)**, comprising taxation, control of availability, and control of advertising. The drink-driving countermeasure was excluded from this scoring analysis. (B) We employed differential weighting for different policy domains (a score of 1 for each of the taxation and the available control policy and a score of 0.5 for the advertising control policy), a single scoring for the policies in place and their implementation. (C) We had two objectives in examining the impacts of policy implementation: (1) to determine the relationship between the year 2020 policy score and the APC for the year 2000, and (2) to determine the relationship between the 2000–2022 policy score and the absolute changes in APC during the same period.

To quantify the availability of alcohol control policies and the timeliness in implementing them, we needed to set scores for these attributes. Since the policy scoring for the second objective analysis covered more details than the first one, we explain the policy scoring for the second objective (analysis of policies implemented from 2000 to 2022) first. We were not only interested in which policies and their detailed measures were implemented between the years 2000 and 2022, but also in how early the respective country implemented them. For countries that were low- income countries (LICs) in 2000 (Cambodia (KHM), Indonesia (IND), Lao PDR (LAO), Myanmar (MMR), and Viet Nam (VNM)), we used the year that the country's income status changed from a LIC to a lower middle-income country (L-MIC) as the reference year. If a particular policy was implemented before this reference year, we considered it to be an early implementation, and considered it to be a late implementation if it was implemented later than this reference year. As a L-MIC in 2000, Thailand's year of economic transition from a L-MIC to an upper middle-income country (U-MIC), 2010, was used as the reference. We used the year 2011 (midpoint of 2000–2022) as the reference year for four countries that did not change their country income status: Brunei Darussalam (BRN: high-income country (HIC)), Singapore (SGP: HIC), Malaysia (MYS: U-MIC), and the Philippines (PHL: L-MIC).

For example, Lao PDR's economic status changed in 2010. The number of years before the reference time was -4 for the minimum legal purchasing age (MLPA) measure implemented in 2014, and it was +2 for the alcohol advertising restriction launched in 2008. For Singapore, the number of years before its reference year of 2011 was -4 for the MLPA implemented in 2015 and +8 for the amendment of the advertising code of conduct in 2003. Therefore, the higher the number of years calculated, the earlier the policy had been implemented.

Since there were huge variations in detailed measures implemented under each policy, we assessed them only on the following characteristics. For taxation, we focused on the tax method and base used, number of tax increases, and the number of these that had taken place before the country's economic transition year or other reference year, inflation-indexing, whether or not the excise taxes were increased between 2000 to 2022 for all alcoholic beverage categories, the percentage of excise tax to the retail price (% tax-to-price), and the percentage of unrecorded to the total alcohol consumption. The tax method and base include the specific tax (taxing an alcohol product based on its ethanol content), the *ad valorem* tax (taxing based on the alcohol price), and the unitary tax (taxing based on the volume of alcoholic beverages). For availability control, we addressed measures restricting age limits, places, and times for both alcohol sales and drinking. For advertising control, we emphasized measures restricting alcohol advertising channels and content. Advertising channels covered television, radio, online media, etc., while advertising content included drinking persuasion or using celebrities or cartoon characters in advertisements, etc., for example.

See **Table S1** for the detailed scoring of each of these three alcohol control policies implemented between 2000 and 2022. The total taxation score (2–19) consisted of the scores of the tax method and base (0–2), the number of tax increases between 2000 and 2022 (0–3) and how early on they had been implemented (1–2), whether or not the tax was indexed to inflation (0–2), whether or not the excise taxes were increased between 2000 and 2022 for all alcoholic beverage categories (0–2), the % tax-to-price (1–4), and the percentage of unrecorded to total alcohol consumption (0–4), combined. The total availability control score (0–12) combined the scores of age limit, place restriction, and time restriction for alcohol sales and drinking. The score of 0–2 was assigned for each of these six availability control measures, resulting in a score of 0–12 in total.

The total advertising control score (0–8) consisted of the score of restriction on advertising channels and content. A score of 0–4 was assigned to each of these two advertising control measures, resulting in a score of 0–8 in total. After standardizing each of these three total scores to be 0–5 for taxation and availability control, and to 0–2.5 for marketing control, as there is much more evidence for immediate effects as well as changes in temporal proximity to the enactment of taxation and availability control policies, than for marketing restrictions **(28)**. We then took the weighted average of them to get a standardized policy score for each country (0–5). Subsequently, we ranked all 10 countries according to their standardized policy score, where a score of 1 denoted the highest score (= most effective policy) and a score of 10 denoted the lowest (= least effective policy).

To incorporate the effect of Islam, which effectively controls alcohol consumption by cultural stigma in Muslim-majority countries, we assigned a "religion" score of 0.05 for each 1% of the population in the country that was predominantly Muslim for each ASEAN country. We then calculated a total for the policy and religion scores (0–10) for each country. Again, we standardized this score to be a maximum of 5, followed by ranking countries based on this score (score of 1 for the highest to a score of 10 for the lowest).

For the second objective (analysis of policies implemented in 2000), Table S1 also shows the detailed scoring for each of these three alcohol control policies implemented in 2000. The scoring structure for the analysis of policies implemented in 2000 is similar to that of the analysis of the year 2000–2022, except for the following. (A) We did not take into account the timeliness of the policy implementation. For example, two categories for whether an age limit for alcohol purchasing was implemented late (a score of 1) or implemented early (a score of 2) were combined into a single category simply denoting if there was an age limit for alcohol purchasing in 2000 or not (a score of 2 was given for having one). (B) We were only concerned with the cross-sectional status of the policies implemented in 2000 only. Hence, policy changes implemented after 2000 were not scored. As a result, the total score of the taxation policy, the availability control policy, and the advertising control policy were changed to 0-4, 0-6, and 0-6, respectively. (C) The religion score was not included. However, the procedures for standardization and weighting were similar. Table S3 demonstrates the policy scores for policies implemented in 2000 among 10 ASEAN countries.

Table S1: Criteria for alcohol control policy scoring

Policy	Policy description	Scale for analysis		Code
		For 2000	For 2000–2022	
**POLICY FACTOR**				
**Taxation**				

Tax method and base	- No excise taxation system with no total ban on alcohol	0	0	a1
	- Applying<br>(a) the unitary tax (taxing volume of an alcoholic beverage) or<br>(b) the *ad valorem* tax (taxing price of an alcoholic beverage)	1	1	
	- Applying<br>(a) the specific tax (taxing ethanol contained in an alcoholic beverage),<br>(b) combination tax (taxing an alcoholic beverage using at least two taxes simultaneously) with the specific tax as a component,<br>(c) multiple taxes applying on different alcoholic beverage types with the specific tax as one of the tax methods used (e.g., employing unitary tax for wine and specific tax for spirits), or<br>(d) total ban on alcohol (e.g. no sales of alcohol or drinks)	2	2	
Number of tax increases between 2000–2022	- No tax increase at all	-	0	a2
	- Tax increases from 1–5 times	-	1	
	- Tax increases from 6–10 times	-	2	
	- Tax increases from >10 times or applying the total ban on alcohol	-	3	
Early tax increase	- Having at least one tax increase with early implementation for at most half of those tax increases	-	1	a3
	- Having at least one tax increase with early implementation for more than half of those tax increases	-	2	
Inflation-Indexing of taxation	- No inflation-indexing	0	0	a4
	- Having inflation-indexing or applying a total ban on alcohol	2	2	
Overall tax increase comparing 2022 to 2000 (y/n)	- Not for some alcoholic beverage categories	-	0	a5
	- Yes, for all alcoholic beverage categories or applying the total ban on alcohol	-	2	
Tax rate (% tax on retail price)	- < 15 %	-	1	a6
	- ≥ 15 % and <30%	-	2	
	- ≥ 30% and < 40%	-	3	
	- ≥ 40% or applying the total ban on alcohol	-	4	
% unrecorded alcohol	- % of the unrecorded to the total alcohol consumption ≥ 50%	-	0	a7
	- % of the unrecorded to the total alcohol consumption between 30% and < 50%	-	1	
	- % of the unrecorded to the total alcohol consumption between 20% and < 30%	-	2	
	- % of the unrecorded to the total alcohol consumption between 10% and < 20%	-	3	
	- % of the unrecorded to the total alcohol consumption between < 10%	-	4	
	**The total taxation score (A-total = a1 + a2 + a3 + a4 + a5 + a6 + a7)**	**0-4**	**2-19**	**A-total**
	**Standardized taxation score (A = standardizing 0-4 and 2-19 to a maximum of 5)**	**0-5**	**0-5**	**A**
**Availability control**				
Age limit	- No control measure at all	0	0	b1
	- Having a measure but late implementation	1	1	
	- Having a measure and early implementation or a total ban on alcohol		2	

Place and time restrictions	The same scores were applied to the age limit for alcohol drinking (b2), place restriction for sale (b3) and drinking (b4), time restriction for sale (b5) and drinking (6)			b2-b6
	**The total availability control score (B-total = b1 + b2 + b3 + b4 + b5 +b6)**	**0-6**	**0-12**	**B-total**
	**Standardized availability control score (B = standardizing 0-12 to a maximum of 5)**	**0-5**	**0-5**	**B**
**Advertising control**				
Channels	- No advertising control measure at all	0	0	c1
	- Alcohol industry self-regulation	1	1	
	- Restricting some advertising channels but late implementation	2	2	
	- Restricting some advertising channels with early implementation		3	
	- Total advertising ban	3	4	
Content	- No advertising control measure at all	0	0	c2
	- Alcohol industry self-regulation	1	1	
	- Restricting some advertising channels but late implementation	2	2	
	- Restricting some advertising channels with early implementation		3	
	- Total advertising ban	3	4	
	**The total advertising control score (C-total = c1 + c2)**	**0-6**	**0-8**	**C-total**
	**Standardized advertising control score (C = standardizing 0-6 and 0-8 to a maximum of 2.5)**	**0-2.5**	**0-2.5**	**C**
	**AVERAGE POLICY SCORE (D = weighted average of A, B, and C)**	**0-5**	**0-5**	**D**
**RELIGION FACTOR**				
% of Muslim	- Each 1% of Muslim population of the country	-	+0.05	e1
**POLICY FACTOR plus RELIGION FACTOR**				
	**The total policy score plus the religion score (E-total = D + e1)**	**N/A**	**0-10**	**E-total**
	**Standardized policy score plus religion score (E = standardizing 0-10 to a maximum of 5)**	**N/A**	**0-5**	**E**
**RANKING**				
	- Ranking from the highest to the lowest score	1-10	1-10	

Apart from unrecorded consumption **(31, 32)**, which can be seen as a measure on the effectiveness of enforcing taxation, we did not take into account the intensity of alcohol control policies or the level of enforcement in the respective countries. We opted not to do this to avoid introducing too much complexity into our assessments. For example, we did not and can not quantify the restriction on alcohol advertising on billboards used in one country and compare it to the restriction on advertising on television between 6 to 8 p.m. in another country and from 10 p.m. to 5 a.m. in yet another country. All these types of alcohol advertising restrictions were classified as partial bans, however. We hypothesized that once the country's government started implementing an alcohol control policy, it would mean alcohol control had moved in a favourable direction in that country. Except for data on unrecorded consumption, there were also no good comparable data on alcohol policy enforcement among the 10 ASEAN countries.

Table S2: Alcohol control policies in 10 ASEAN countries in 2000–2022

Country		Alcohol control policy description	Summary for Table 3	Score for Table 4	Score for Table S3
**Brunei Darussalam**					
Taxation	Tax method	- Prohibit alcohol sale	- Prohibit alcohol sale	2	2
	Number of tax increases	- Prohibit alcohol sale	- Prohibit alcohol sale	3	-
	Early tax increase	- Prohibit alcohol sale	- Prohibit alcohol sale	2	-
	Inflation-indexing	- Prohibit alcohol sale	- Prohibit alcohol sale	2	0
	Overall tax increases comparing 2022 to 2000	- Prohibit alcohol sale	- Prohibit alcohol sale	2	-
	% tax-to-price	- Prohibit alcohol sale	- Prohibit alcohol sale	4	-
	% of unrecorded alcohol	- 21.8%	- 21.8%	2	-
Availability control	Age	- 1962: Prohibit alcohol drinking (based on Islamic Law)<br>- 1990: Prohibit alcohol sales	- Prohibit alcohol drinking (+49)<br>- Prohibit alcohol sale (+21)	4	2
	Place			4	2
	Time			4	2
Advertising control		- 2000: Prohibit alcohol advertising	- Prohibit alcohol advertising or promotion (+11) [both advertising channels and content]	8	6
Drink-driving control		- 1984: BAC 80 mg%; breath alcohol concentration 35 mg in 100 ml of breath (as stipulated in Road Traffic Act (1984))	- BAC 80 mg% (+16)	N.A.	N.A.
**Indonesia**					
Taxation	Tax method	- Unitary tax (taxing based on the volume of alcoholic beverage)	- Unitary tax	1	1
	Number of tax increases	- Alcohol tax increases in 2006 by 25-100%, in 2010 by 100-300%, and in 2014 by 6.9-50%	- Three tax increases	1	-
	Early tax increase		- Zero times before the 1st economic transition	1	-
	Inflation-indexing	- No inflation-indexing for alcohol taxation	- No	0	0

Country		Alcohol control policy description	Summary for Table 3	Score for Table 4	Score for Table S3
	Overall tax increases comparing 2022 to 2000	- Yes	- Yes	2	-
-	% tax-to-price	- 22.1% for beer, 40.0% for spirits	- 22.1% for beer, 40.0% for spirits, 27.0% for weighted average	2	-
-	% of unrecorded alcohol	- 35.1%	- 35.1%	1	-
Availability control	Age	- 2014: Minimum legal purchasing age of 21 years old	- 21 (-12 [year]) (for sale)	1	0
	Place	- 2014: restrict alcohol sale venues (e.g. hotel), locations (e.g., not allow to sell alcohol close to youth centre), point-of-sale (e.g., separate alcohol sale from other products)<br>- 2015: additionally prohibit alcohol sale in minimarkets	- ✓ (-12) (for sale)	1	0
	Time	-	-	0	0
Advertising control		- 2002: prohibit alcohol advertising via broadcasters<br>- 2014: prohibit alcohol advertising via alcohol industry<br>- Alcohol advertising at sale venues is not prohibited.	- P (+0) (for channel restriction) [P = partial ad ban)<br>- P (-12) [This does not count as late implementation because it followed the 2002 policy intervention.	3	2
Drink-driving control		- 2009: fines for driving carelessly; however, no illegal blood alcohol concentration limit (BAC) is determined.	- Careless driving (-7)	N.A.	N.A.
**Malaysia**					
Taxation	Tax method	- Specific tax (taxing based on the 100% volume per litre)	- Specific tax	2	2
	Number of tax increases	- Excise tax increases in 2004, 2012, 2016, 2017, and 2022	- five tax increases between 2000 and 2022	1	-
	Early tax increase		- Once before 2011	1	-
	Inflation-indexing	- No inflation-indexing for alcohol taxation	- No	0	0
	Overall tax increases comparing	- Yes	- Yes	2	-

Country		Alcohol control policy description	Summary for Table 3	Score for Table 4	Score for Table S3
	2022 to 2000				
	% tax-to-price	- 31.1% for beer, 15.6% for spirits	- 31.1% for beer, 15.6% for spirits<br>- Weighted average 28.0%	2	-
	% of unrecorded alcohol	- 23.5%	- 23.5%	2	-
Availability control	Age	- 1985: Minimum legal purchasing age of 21 years old for buying alcoholic beverages, based on Food Regulations 1985	- 21 (+26 [year]) (for sale)	2	1
	Place	-	-	0	0
	Time	- 1977: prohibit alcohol sale from 9 p.m. to 7 a.m.	- 10 (+34 [year]) (for sale)	2	1
Advertising control		- 1994: prohibit alcohol advertising via broadcasts and billboards<br>- 2022: allow ads in electronic media, but restrict their content to non-Muslim audiences aged ≥21	- P (+17) (for channel restriction) [P = partial ad ban)<br>- P (-11)	3	2
Drink-driving control		- 1976: BAC 80 mg%, 35 mg of alcohol in 100 ml of breath, 107 ml of alcohol in 100 ml of urine<br>- 2009: BAC 50 mg%, 22 mg of alcohol in 100 ml of breath, 67 ml of alcohol in 100 ml of urine	- 80 mg% (+35), 50 mg% (+2)	N.A.	N.A.
**Philippines**					
Taxation	Tax method	- 2004: tax on spirits (specific tax), on wine and fermented (unitary tax), tiers based on price<br>- 2012: tax on spirits (mixed *ad valorem* and specific tax), fermented liquors (unitary tax), inflation-indexing (4%/yr)<br>- 2017: 0% duty for alcohol from AUS/NZ<br>- 2020: increase tax and indexing inflation to 6% by 2024	- Multiple taxes, based on specific, *ad valorem* and unitary taxes	2	2
	Number of tax increases		- 12 tax increases between 2000 and 2022, including tax increases due to inflation-indexing<br>-	3	-
	Early tax increase	- One tax increase in 2004	- One tax increase before 2011	1	-
	Inflation-indexing	- 2012: inflation-indexing (4%/yr)<br>- 2020: indexing inflation to 6% by 2024	- ✓ (-1)	2	0
	Overall tax increases comparing	- Yes	- Yes	2	-

Country		Alcohol control policy description	Summary for Table 3	Score for Table 4	Score for Table S3
	2022 to 2000				
	% tax-to-price	- 30.1% for beer, 44.0% for spirits	- 30.1% for beer<br>- 44.0% for spirits<br>- 41.1% for weighted average	4	-
	% of unrecorded alcohol	- 19.6%	- 19.6%	3	-
Availability control	Age	- No	- No	0	0
	Place	- 2011: prohibiting alcohol drinking in government and university places and drinking by government staff on duty	- ✓ (+0) for drinking (in some public places)	1	0
	Time	- No	- No	0	0
Advertising control		- Before 2000: Industrial self-regulation	- Before 2000: Industrial self-regulation	1	1
Drink-driving control		- 2002: 50 mg% for general drivers, 10 mg% for professional drivers and motorcyclists	- 50/10 mg% (+9)	N.A.	N.A.
**Singapore**					
Taxation	Tax method	- Specific tax (taxing based on the ethanol contained in an alcoholic beverage)	- Specific tax	2	2
	Number of tax increases	- 2002: excise tax increase<br>- 2004: excise tax increase<br>- 2014: taxing alcohol preparations at $88/LPA (litre of pure alcohol) for spirits and wines, and $60/LPA for beer (with additional $16/LPA for imported beer) (based on last effective duty raise in 2004. To note: the 2014 tax increase was a 25% increase on beer, wine, and spirits to account for inflation since 2004)	- Three tax increases between 2000 and 2022 (2002, 2004, 2014)	1	-
	Early tax increase	- Two tax increases in 2002 and 2004	- More than half of tax increases occurred before 2011	2	-
	Inflation-indexing	- No inflation-indexing for alcohol taxation	- No	0	0
	Overall tax increases comparing 2022 to 2000	- Yes	- Yes	2	-
	% tax-to-price	- 31.0% for beer, 36.9% for imported beer, 30.5% for spirits	- 31.0% for beer, 30.5% for spirits<br>- Weighted average 30.9%	3	-

Country		Alcohol control policy description	Summary for Table 3	Score for Table 4	Score for Table S3
	% of unrecorded alcohol	- 9.6%	- 9.6%	4	-
Availability control	Age	- before 2000: Minimum legal purchasing and drinking age of 18 years old	- 18 (+11 [year]) for sale<br>- 18 (+11 [year]) for drinking	4	2
	Place	- 2015: restrict alcohol drinking in some public places on weekends, public holidays; and evenings of public holidays, not allowed during the hours of 7 p.m. to 7 a.m.<br>- restrict online alcohol sales to minors aged < 18 years old	- ✓ (-4) for drinking (in some public places)<br>- ✓ (-4) for sale (through online for minor aged <18 years old)	2	0
	Time	- 1958: public entertainment establishments that serve alcohol, such as bars, nightclubs, and pubs may operate: A) up to 1 a.m. every Monday to Saturday and 2 a.m. every Sunday and public holiday, OR B) up to 3 a.m. every Monday to Saturday and 4 a.m. every Sunday and public holiday; OR c) up to 6 a.m. daily, depending on license<br>- 2015: covers all other alcohol-selling establishments not classified as "public entertainment establishments." Restrict alcohol sales for on-premises consumption between 10 p.m. OR 11:59 p.m. and 6 a.m., depending on license; restrict alcohol sales for off-premises consumption (retail) between 10:30 p.m. and 7 a.m.<br>- 2015: ban on consumption in all public places (beyond the specific spaces listed above) from 10:30 p.m. to 7 a.m.	- ✓ (-4) for off-premise selling<br>- ✓ (-4) for drinking (in public places)	2	0
Advertising control		- 2003: advertising code of conduct and 2008: amendment of code of conduct (based on 1976 code of conduct)	- S (+36, +8, +3) (for self-regulation using the code of conduct)	1	1
Drink-driving control		- 1961: Road Traffic Act 1961 with BAC 80 mg%<br>- 2020: amended Road Traffic Act 1961 to have more penalty for subsequent convictions (BAC = 80 mg%)	- 80 mg% (+50)<br>- More penalty (-9)	N.A.	N.A.
**Thailand**					

Country		Alcohol control policy description	Summary for Table 3	Score for Table 4	Score for Table S3
Taxation	Tax method	- Before 2013: combination tax, namely the Two-Chosen-One tax method;<br>- 2013–2017: combination tax, namely the Two-Chosen-One tax method, under the mixed *ad valorem* and specific tax;<br>- 2017 onward: combination tax, namely the mixed *ad valorem* and specific tax.	- Combination tax based on specific tax	2	2
	Number of tax increases	- tax increases in 2000/1/3/5/7/9/12<br>- 2013: 1st tax restructure<br>- 2017: 2nd tax restructure	- 9 tax changes (seven tax increases plus two tax structural changes) between 2000 and 2022	2	-
	Early tax increase	- Six tax increases before 2010	- More than half of tax increases occurred before 2010	2	-
	Inflation-indexing	- No inflation-indexing for alcohol taxation	- No	0	0
	Overall tax increases comparing 2022 to 2000	- Yes	- Yes	2	-
	% tax-to-price	- 32.2% for beer, 34.2% for spirits	- 32.2% for beer<br>- 34.2% for spirits<br>- 33.6% for weight average	3	-
	% of unrecorded alcohol	- 15.4%	- 15.4%	3	-
Availability control	Age	- 1972: MLPA 18 years old<br>- 2008: MLPA 20 years old<br>- 2003: MLDA 18 years old	- 18 (+7) and 20 (+2 [year]) for sale<br>- 18 (+7 [year]) for drinking	4	2
	Place	- 2008: restrict alcohol sale and drinking in some public places	- ✓ (+2) for sale<br>- ✓ (+2) for drinking	4	0
	Time	- 1972: restrict alcohol sale between 2-5 p.m. and midnight to 11 a.m.	- 14 (+50) for sale	2	1
Advertising control		- 2003: prohibiting advertising on TV and radio between 10 p.m. and 5 a.m.<br>- 2008: restrict alcohol advertising content and alcohol sale promotion	- P (+7) (for channel restriction) [P = partial ad ban)<br>- P (+2) (for content restriction)	6	0
Drink-driving control		- 1994: BAC 50 mg% for all drivers<br>- 1994: BAC 20 mg% for (1) drivers under the age of 20, (2) drivers holding a temporary driver's license, (3) drivers with a license for a vehicle type that cannot be substituted for another, or (4)	- 50 mg% (+16)<br>- 20 mg% for young (+3)	N.A.	N.A.

Country		Alcohol control policy description	Summary for Table 3	Score for Table 4	Score for Table S3
		drivers without a driver's license or those whose license is suspended or revoked			
**Cambodia**					
Taxation	Tax method	- *Ad valorem* tax (taxing based on alcohol price)	- *Ad valorem* tax	1	1
	Number of tax increases	- 2014: tax increase from 10% to 20%<br>- 2016: tax increase from 20 to 35% for alcohol products, and 25 to 30% for beer<br>- 2018: imports from 15 to 35%<br><br>(2024: Public light tax: from 3 to 5%)	- Three tax increase between 2000–2022	1	-
	Early tax increase	- One tax increase happened before the economic transition in 2015	- one tax increase occurred before the economic transition	1	-
	Inflation-indexing	- No inflation-indexing for alcohol taxation	- No	0	0
	Overall tax increases comparing 2022 to 2000	- Yes	- Yes	2	-
	% tax-to-price	- 14.4% for beer<br>- 12.8% for spirits, imputed by using an average ratio of beer to spirits of other nine countries	- 14.4% for beer<br>- 12.8% for spirits<br>- 14.3% for weighted average	1	-
	% of unrecorded alcohol	- 14.2%	- 14.2%	3	-
Availability control	Age	- No	- No	0	0
	Place	- No	- No	0	0
	Time	- No	- No	0	0
Advertising control		(2024: partial advertising control with many measures)	- No advertising control between 2000 and 2022	0	0
Drink-driving control		- 2006: BAC 50/80 mg% (with only fined and imprisoned, respectively)<br>- 2015: increased penalty	- 50/80 mg% (+9)<br>- More penalty (+0)	N.A.	N.A.
**Laos PDR**					
Taxation	Tax method	- *Ad valorem* tax (taxing based on alcohol price)	- *Ad valorem* tax	1	1
	Number of tax increases	- 2005: 70% (for alcohol products >15%), 60% (for alcohol products <15%), 50% (for beer)	- Two tax increase between 2000 and 2022	1	-

Country		Alcohol control policy description	Summary for Table 3	Score for Table 4	Score for Table S3
		- -2021: same for alcohol, 70-80% for Whisky>20%, 60% for beer			
-	Early tax increase	- One tax increase happened before the economic transition (2005)	- One tax increase happened before the economic transition (not more than half)	1	-
	Inflation-indexing	- No inflation-indexing for alcohol taxation	- No	0	0
	Overall tax increases comparing 2022 to 2000	- Yes	- Yes	2	-
	% tax-to-price	- 37.1% for beer, 44.8% for spirits	- 37.1% for beer, 44.8% for spirits<br>- 40.9% for weighted average	4	-
	% of unrecorded alcohol	- 35.6%	- 35.6%	1	-
Availability control	Age	- 2014: MLPA / MLDA 18 years old	- 18 (-4 [year]) for sale<br>- 18 (-4 [year]) for drinking	2	0
	Place	- 2014: restrict on/off alcohol sale venues and gas station<br>- 2014: restrict alcohol drinking in some public	- ✓ (-4) for sale<br>- ✓ (-4) for drinking	2	0
	Time	- 2014: allow alcohol sale from 4 a.m. to 11 p.m.	- 17 (-4) for sale	1	0
Advertising control		- 2008: banned restriction on alcohol ad (based on Law on Media 2008)<br>- 2014: prohibit sponsorship towards minors	- P (+2) (for channel restriction) [P = partial ad ban)<br>- P (-4) (for content restriction)	3	0
Drink-driving control		- 2000: BAC 80 mg%<br>- 2013: BAC 50 mg% (24 mg of alcohol in 100 ml of breath) for 4-wheel drivers, 0 mg% for truck/bus/van drivers, and 30 mg% for 2- or 3-wheel drivers	- 80 mg% (+10)<br>- 50/0/30 mg% (-3)	N.A.	N.A.
**Myanmar**					
Taxation	Tax method	- *Ad valorem* tax (taxing based on alcohol price)	- *Ad valorem* tax	1	1
	Number of tax increases	- 2015: alcohol tax increases (as commercial tax) by 50-60% for beer and spirits<br>- 2016: the minimum tax rate (as specific good tax) for alcohol increased; however, the taxes of 50% for imported	- Two tax increases between 2000 and 2022	1	-

Country		Alcohol control policy description	Summary for Table 3	Score for Table 4	Score for Table S3
		wines and 60% for imported spirits were removed.			
	Early tax increase	- Zero tax increase occurred before 2014	- Zero tax increase occurred before the economic transition	1	-
	Inflation-indexing	- No inflation-indexing for alcohol taxation	- No	0	0
	Overall tax increases comparing 2022 to 2000	- No, for some alcohol categories, which were imported wine and imported spirits	- No for some alcohol categories, which were imported wine and imported spirits	0	-
	% tax-to-price	- 52.5% for beer, 28.1% for spirits (based on WHO (2023))	- 52.5% for beer<br>- 28.1% for spirits<br>- 34.7% for weighted average	3	-
	% of unrecorded alcohol	- 9.5%	- 9.5%	4	-
Availability control	Age	- 1917: Burma Excise Act restricted minimum age of 18 years old for sales of alcoholic beverages	- 18 (+97)	2	1
	Place	- 2013: restrict imported alcohol to be sold only at hotels and duty-free shops (2015: delegate importation of wine)	- ✓ (+1) for sale	2	0
	Time	- 1917: Burma Excise Act restricted alcohol sale between 11 p.m. until sunrise the next day	- ✓ (+97) for sale	2	1
Advertising control		- 2002: ban on alcohol advertising on billboards	- P (+12) (for channel restriction) [P = partial ad ban)<br>-	3	0
Drink-driving control		- 2015: BAC 70 mg% for general drivers, 0 mg% for young and professional drivers	- 70/0 mg% (-1)	N.A.	N.A.
**Viet Nam**					
Taxation	Tax method	- *Ad valorem* tax (taxing based on alcohol price)	- *Ad valorem* tax	1	1
	Number of tax increases	- Alcohol <20%: 25% (2010), 30% (2016), 35% (2018)<br>- Alcohol ≥20%: 45% (2010), 50% (2013), 55% (2016), 60% (2017), 65% (2018)	- Nine tax increases between 2000 and 2022 , including three times involving tax decreases for some alcoholic beverage categories; counted as six instances of tax increases	2	-
	Early tax increase	- No tax increase occurred before 2009	- No tax increase occurred before the economic transition	1	-

Country		Alcohol control policy description	Summary for Table 3	Score for Table 4	Score for Table S3
	Inflation-indexing	- No inflation-indexing for alcohol taxation	- No	0	0
	Overall tax increases comparing 2022 to 2000	- No for more than 40% ABV spirits, bottled and draft beer	- No for more than 40% ABV spirits, bottled and draft beer	0	-
	% tax-to-price	- 28.7% for beer, 28.7% for spirits	- 28.7% for beer, 28.7% for spirits<br>- 28.7% for weighted average	2	-
	% of unrecorded alcohol	- 71.5%	- 71.5%	0	-
Availability control	Age	- 2020: MLPA/MLDA 18 years old	- 18 (-11 [year]) for sale<br>- 18 (-11 [year]) for drinking	2	0
	Place	- 2020: restrict alcohol sale and drinking in some places and locations	- ✓ (-11) for sale<br>- ✓ (-11) for drinking	2	0
	Time	- No	- No	0	0
Advertising control		- 2020: alcohol advertising ban on alcohol > 15%, and partial advertising ban on alcohol < 15% (including no advertising at 6-9 p.m.)	- P (-11) (for channel restriction) [P = partial ad ban)<br>- P (-11) (for content restriction)	4	0
Drink-driving control		- 2020: BAC 0 mg%	- 0 mg% (-11)	N.A.	N.A.

Table S3. Alcohol control policy enactments and timeliness among 10 ASEAN countries during 2000–2022

	Policy Descriptors	BRN††	IDN††	MYS††	PHL‡‡	SGP‡‡	THA‡‡	KHM§§	LAO§§	MMR§§	VNM§§
**Taxation**											
Tax method		Ban on sales of alcohol	Unitary	Specific	Multiple	Specific	Combination tax	*Ad valorem*	*Ad valorem*	*Ad valorem*	*Ad valorem*
Title Tax base	Ethanol		-	-	✓	✓	✓	-	-	-	-
	Beverage volume		✓	✓	✓	-	-	-	-	-	-
	Price		-	-	✓	-	✓	✓	✓	✓	✓
Number of tax increases	2000–2022		3	5	12	3	9*	3	2	2	5
	Before the reference time†		0	1	1	2 (more than half)	6 (more than half)	1	1	0	0
Inflation indexing			-	-	✓ (−1)	-	-	-	-	-	-
Overall tax increase comparing 2022 to 2000			Yes	Yes	Yes	Yes	Yes	Yes	Yes	No for imported spirits and wine	No for more than 40% ABV spirits, bottled and draft beer
% of tax-to-price [33]	Beer		22.1	31.1	30.1	31.0	32.2	14.4	37.1	52.5	28.7
	Spirits		40.0	15.6	44.0	30.5	34.2	12.8†	44.8	28.1	28.7
	Weighted average§		27.0	28.0	41.1	30.9	33.6	14.3	40.9	34.7	28.7
% of unrecorded alcohol		21.8	35.1	23.5	19.6	9.6	15.4	14.2	35.6	9.5	71.5
**Availability control**											

Age limit in years (number of years before)¶	Sale	Ban on public drinking (+49) and sales (+21); non-Muslims may import limited quantities for use in private settings	21 (−12)	21 (+26)‖	-	18 (+11)	18 (+7), 20 (+2)	-	18 (−4)	18 (+97)	18 (−11)
	Drink		-	-	-	18 (+11)	18 (+7)	-	18 (−4)	-	18 (−11)
Place restriction (number of years before)	Sale		✓ (−12)	-	-	✓ (−4)	✓ (+2)	-	✓ (−4)	✓ (+1)	✓ (−11)
	Drink		-	-	✓ (+0)	✓ (−4)	✓ (+2)	-	✓ (−4)	-	✓ (−11)
Time restriction (number of years before)	Sale		-	10 (+34)	-	✓ (−4)	14 (+50)	-	17 (−4)	✓ (+97)	-
	Drink		-	-	-	✓ (−4)	-	-	-	-	-
**Advertising control**											
Channel restriction (number of years before)		Total ad ban (+11) for both channel and content	P (+0), P (−12)	P (+17), P (−11)	-	-	P (+7)	-	P (+2), P (−4)	P (+12)	P (−11)

Content restriction (number of years before)			-	-	S (+11)	S (+36, +8, +3)	P (+2)	-	-	-	P (−11)
**Drink-driving control**											
BAC in mg% (number of years before)		80 mg% (+16)	Driving carelessly (−7)	80 (+35), 50 (+2)	50/10** (+9)	80 mg% (+50), more penalty (−9)	50 (+16), 20 for young (+3)	50/80** (+9), more penalty (+0)	80 (+10), 50/0/30 (−3)	70/0** (−1)	0 (−11)

ABV – alcohol by volume, APC – alcohol *per capita* consumption, ASEAN – Association of Southeast Asian Nations, BAC – blood alcohol concentration, BRN – Brunei, HIC – high-income country, IDN – Indonesia, KHM – Cambodia, LAO – Lao PDR, LIC – low-income country, LMIC – lower middle-income country, MMR – Myanmar, MYS – Malaysia, PHL – Philippines, SGP – Singapore, THA – Thailand, UMIC – upper middle-income country, VNM – Viet Nam

* The nine changes to laws include seven tax increases plus two tax structural changes.

†Number of tax increases before economic transition for countries having economic transition or before the year 2011 (mid of 2000-2022) for countries having no economic transition.

‡Cambodia's estimate for spirits was imputed using an average ratio of beer to spirits of other nine countries.

§Weighted average was calculated by taking into account the market shares of beer and spirits.

¶Number of years before: number of years prior to economic transition for countries having an economic transition, or number of years prior to the year 2011 (middle of 2000–2022) for countries having no economic transition.

‖Minimum legal purchasing age is 21 y old for buying alcoholic beverages, based on Food Regulations 1985.

**For Philippines: 50 mg% for general drivers and 10 mg% for professional drivers and motorcyclists. For Cambodia: >50 mg% leads to fine penalty and >80 mg% to imprisonment penalty. For Lao PDR: 50 mg% for four-wheel drivers, 0 mg% for truck/bus/van drivers, and 30 mg% for two- or three-wheel drivers. For Myanmar: 70 mg% for general drivers and 0 mg% for young and professional drivers.

††Cluster 1

‡‡Cluster 2

§§Cluster 3

Table S4: Scores and ranking of alcohol control policies, religion factors, and APC for 10 ASEAN countries in 2000

Policy Score (Score Criteria)	Code from Table 3	BRN	IDN	MYS	PHL	SGP	THA	KHM	LAO	MMR	VNM
ALCOHOL CONTROL POLICY											
Tax score		4	1	2	2	2	2	1	1	1	1
**Standardized taxation score (0-5)**	**A**	**5.00**	**1.25**	**2.50**	**2.50**	**2.50**	**2.50**	**1.25**	**1.25**	**1.25**	**1.25**
Availability control		6	0	2	0	2	3	0	0	2	0
**Standardized availability control score (0-5)**	**B**	**5**	**0.00**	**1.67**	**0.00**	**1.67**	**2.50**	**0.00**	**0.00**	**1.67**	**0.00**
Advertising control score		6	2	2	1	1	0	0	0	0	0
**Standardized advertising control score (0-2.5)**	**C**	**2.50**	**0.83**	**0.83**	**0.42**	**0.42**	**0.00**	**0.00**	**0.00**	**0.00**	**0.00**
**Weighted average standardized policy score (0-5)**		**5.00**	**0.83**	**2.00**	**1.17**	**1.83**	**2.00**	**0.50**	**0.50**	**1.17**	**0.50**
**Ranking policy scores**	**D**	**1**	**7**	**2**	**5**	**4**	**2**	**8**	**8**	**5**	**8**
**APC absolute in 2000[a]**		**0.34**	**0.09**	**0.71**	**5.85**	**2.12**	**8.30**	**0.80**	**8.63**	**0.26**	**3.88**
**Ranking APC in 2000 (1 = least APC)**		**3**	**1**	**4**	**8**	**6**	**9**	**5**	**10**	**2**	**7**

Abbreviations: APC: alcohol *per capita* consumption, BRN: Brunei Darussalam, IDN: Indonesia, KHM: Cambodia, LAO: Lao PDR, LPA: litre of pure alcohol, MMR: Myanmar, MYS: Malaysia, PHL: Philippines, SGP: Singapore, THA: Thailand, VNM: Viet Nam

[a] Data from **Table 1**

Appendix S2

Figure S1: Overview of wealth in GDP-PPP *per capita* (in $ Int.), adult alcohol *per capita* consumption (APC), and life expectancy at birth in all 10 ASEAN Member States

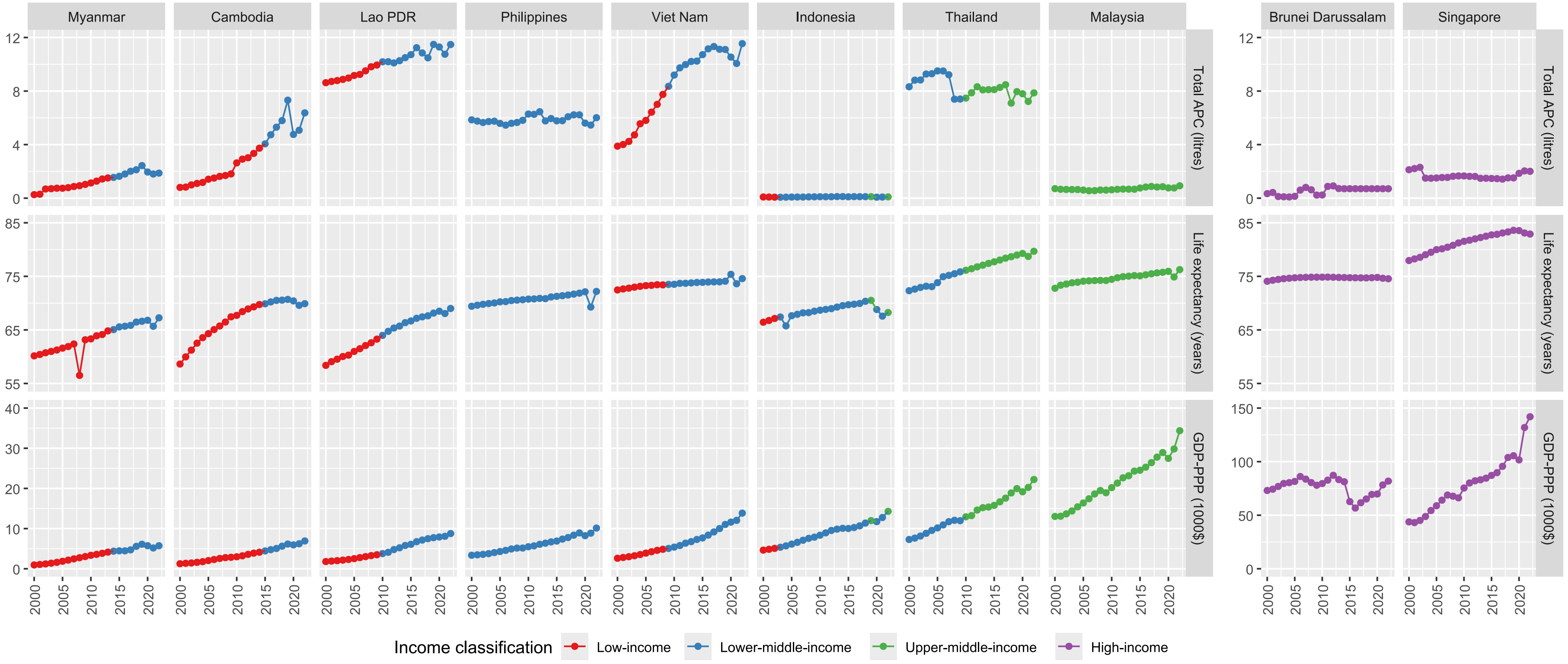

Figure S2: Overview of wealth in GDP-PPP *per capita* (in $ Int.), adult alcohol *per capita* consumption (APC), and life expectancy at birth for the ASEAN region and by cluster, weighted by population size

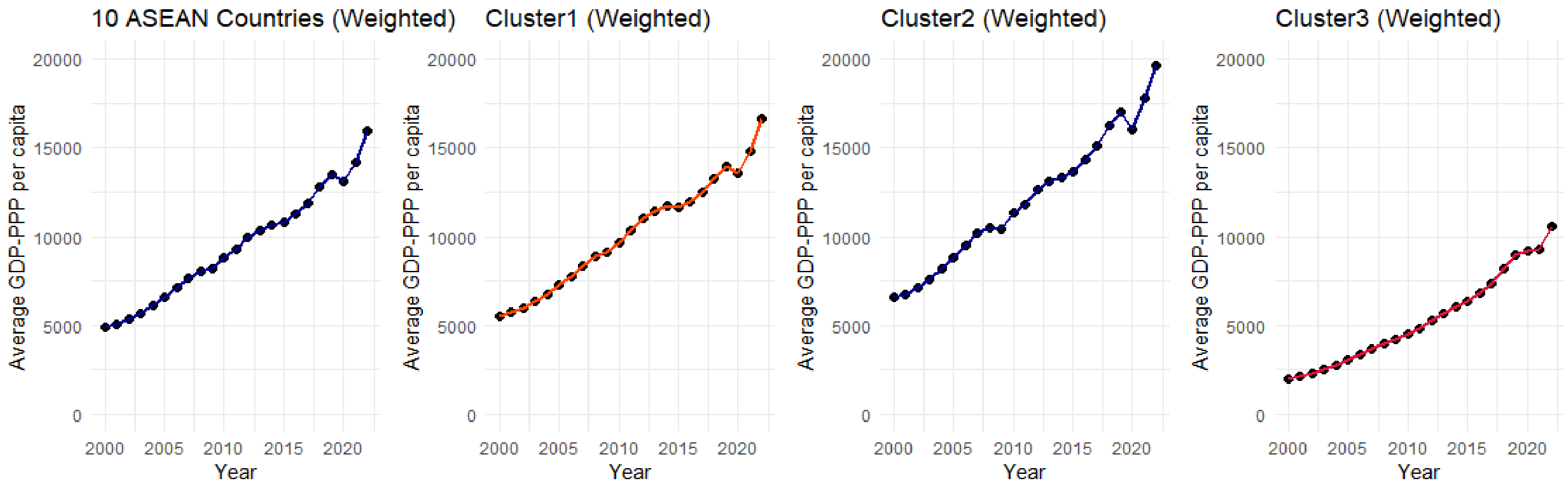

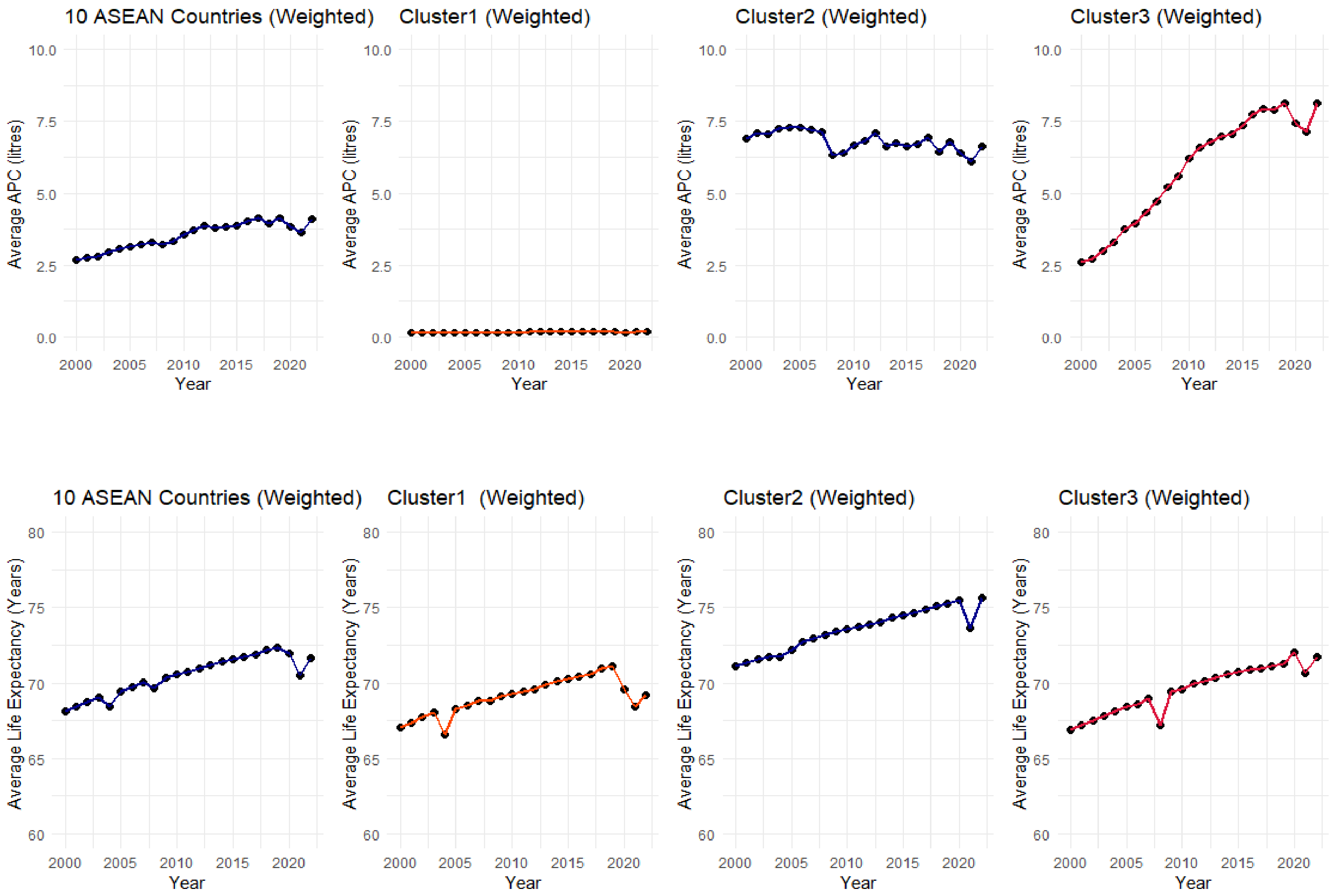
10 ASEAN Countries (Weighted)
Cluster1 (Weighted)
Cluster2 (Weighted)
Cluster3 (Weighted)
Average APC (litres)
0.0
2.5
5.0
7.5
10.0
2000
2005
2010
2015
2020
Year
10 ASEAN Countries (Weighted)
Cluster1 (Weighted)
Cluster2 (Weighted)
Cluster3 (Weighted)
Average Life Expectancy (Years)
60
65
70
75
80
2000
2005
2010
2015
2020
Year

Appendix S3: Cluster analysis based on trajectories

This Appendix provides further details on the clustering analysis conducted to group countries according to their trajectories in three key indicators: adult (15+) alcohol *per capita* consumption (APC) in litres of pure alcohol, gross domestic product *per capita* at purchasing power parity (GDP-PPP) in $ 1,000 Int, and life expectancy in years, over the period 2000–2022. The goal of this analysis was to identify similarities in temporal trends across countries, which may provide a useful tool for comparing countries with similar developmental patterns and contextualizing their alcohol control policy frameworks.

Linear regression models were fitted for each country and variable to estimate the trajectory over time. The resulting intercept and slope coefficients (2 per variable, 6 total per country) were used to summarize the level and direction of change for each country across the three indicators. The trajectories are described in Figure S3.

Figure S3: Estimated trajectories of APC, GDP-PPP, and life expectancy from 2000 to 2022 for each of the ten countries. Solid lines represent the linear regression fit used to derive intercept and slope coefficients for the clustering analysis

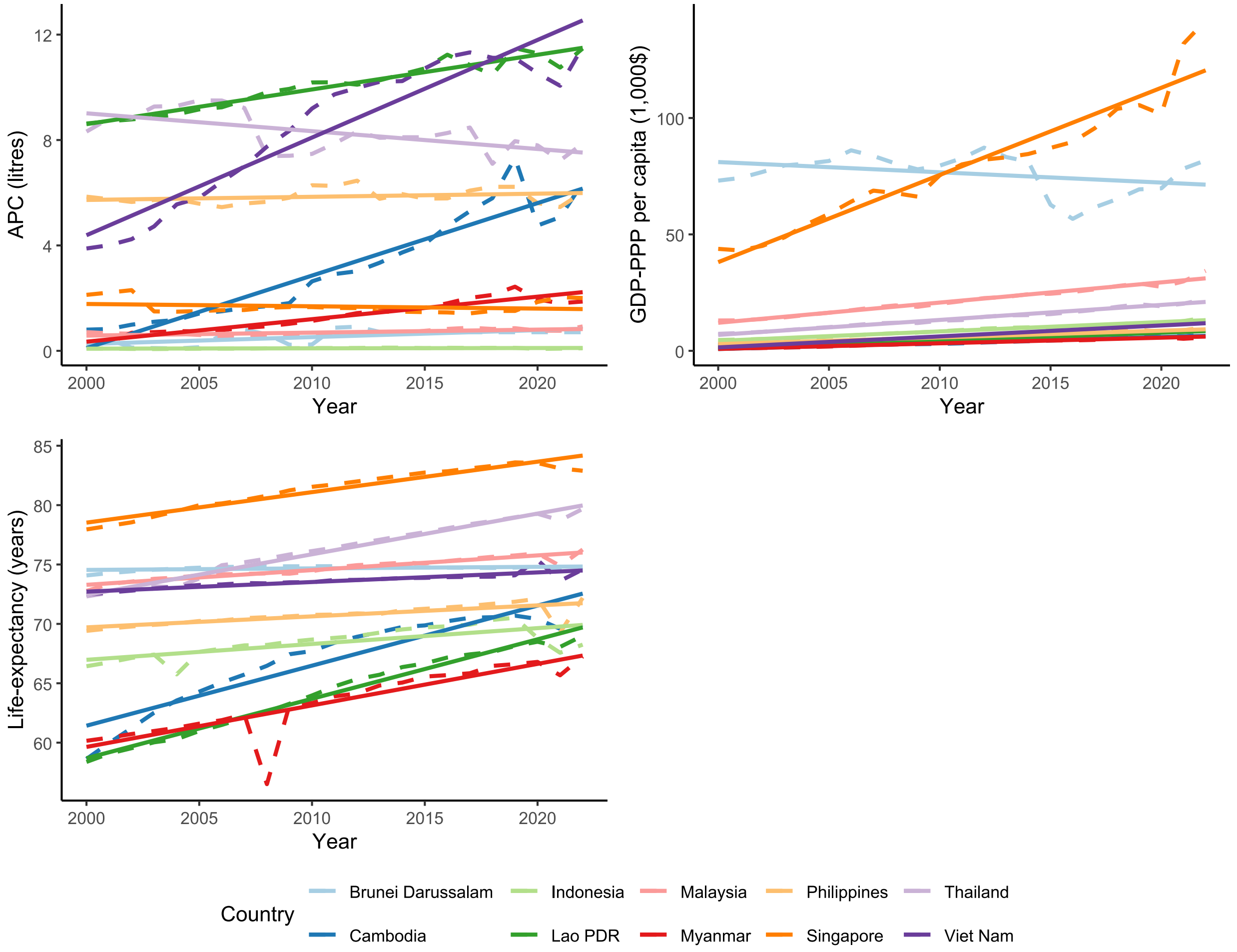

Hierarchical clustering was applied to the dataset with six variables (intercept and slope for each of the three variables). The Euclidean distance metric was used to compute dissimilarities between countries, and clusters

were formed using Ward's minimum variance method. To determine the optimal number of clusters, both visual inspection of the dendrogram (Figure S4) and the elbow method (Figure S5) were used.

Figure S4: Dendrogram from hierarchical clustering based on six trajectory coefficients per country.

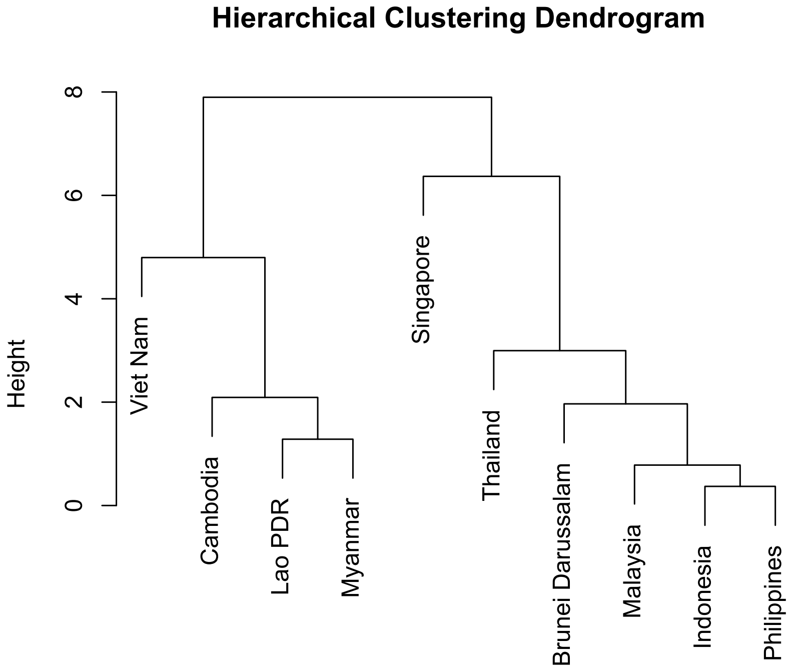

Figure S5: Elbow method plot for identifying the optimal number of clusters.

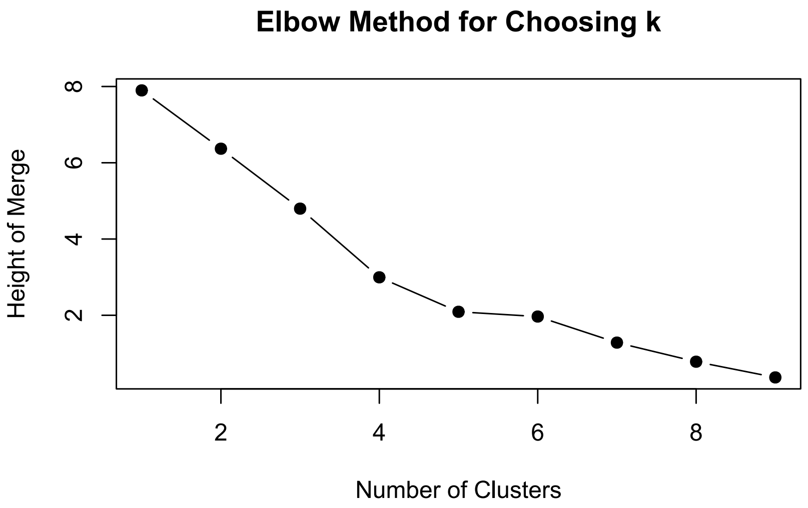

The hierarchical clustering identified two main clusters based on the trajectories of APC, GDP-PPP, and life expectancy over the 2000–2022 period. The first cluster included KHM, LAO, MMR, and VNM, while the second

cluster comprised BRN, IDN, MYS, PHL, SGP, and THA. These groupings are illustrated in Figure S6, which shows the evolution of APC, GDP-PPP, and life expectancy from 2000 to 2022, colored by cluster assignment. Countries in the first cluster were characterized by relatively stable levels of APC, higher GDP-PPP, and higher life expectancy. The second cluster included countries with increasing trends in APC, alongside lower GDP-PPP and lower life expectancy.

Figure S6: Countries grouped by the two-cluster solution, showing the trajectories of APC, GDP-PPP, and life expectancy from 2000 to 2022. Each line represents a country, colored by cluster assignment.

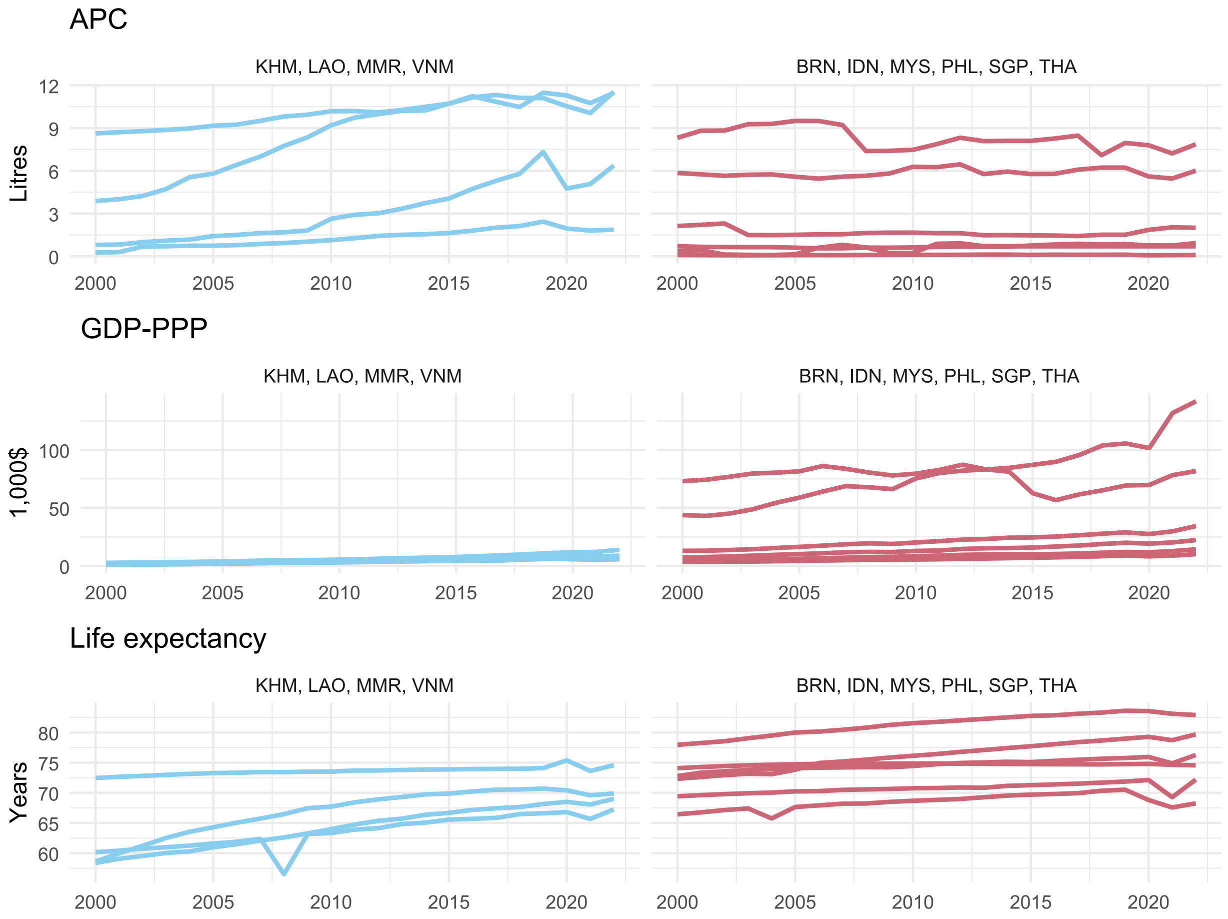

Although the dendrogram suggested a natural division into two groups, the elbow method indicated that a five-cluster solution would better capture heterogeneity in the data. This finer-grained solution retained the two initial clusters and identified three countries—VNM, SGP, and THA—as distinct one-country clusters due to their unique trajectories. The resulting five-cluster solution is shown in Figure C5.

Figure S7: Countries grouped by the five-cluster solution, showing the trajectories of APC, GDP-PPP, and life expectancy from 2000 to 2022. Each line represents a country, colored by cluster assignment

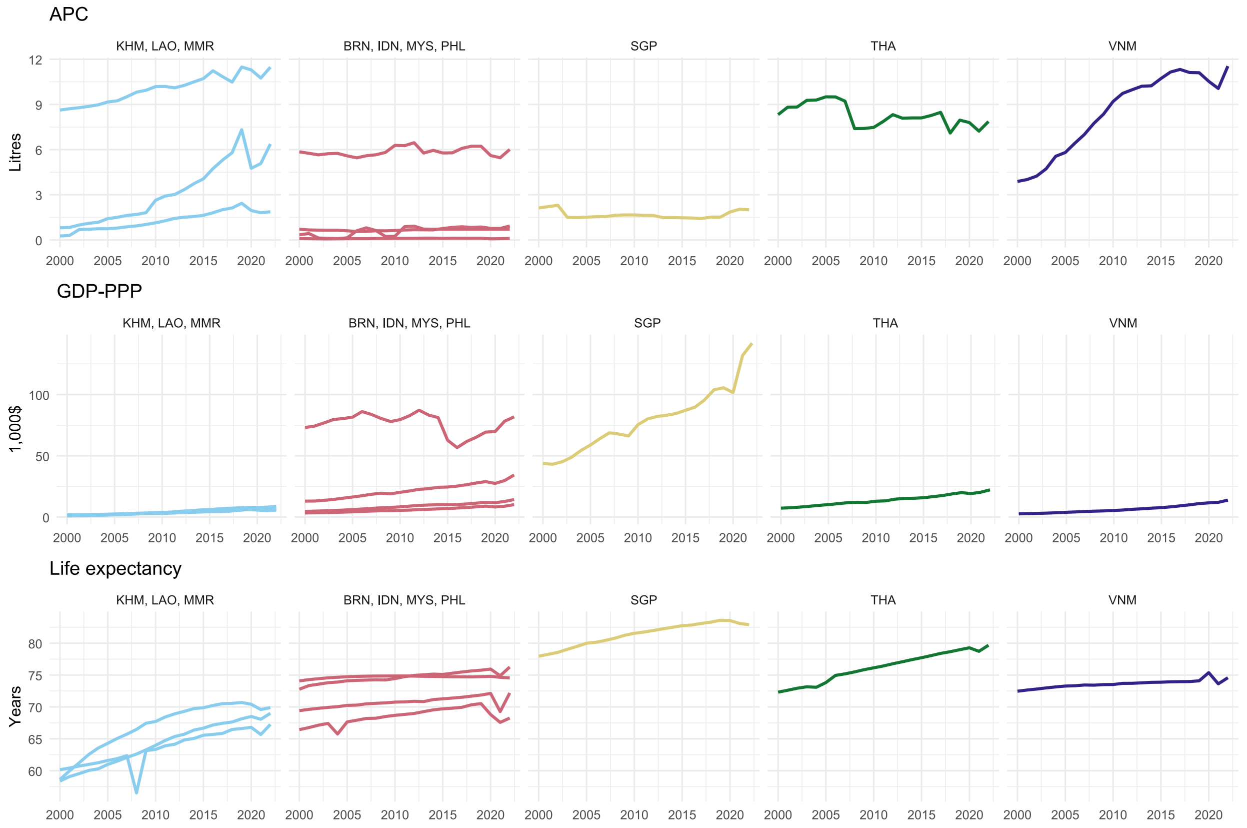

Appendix S4: Association between GDP-PPP *per capita* and life expectancy and population alcohol consumption (APC)

Pearson correlations

Table S4: Pearson correlations between GDP-PPP *per capita* and life expectancy at birth, and APC 2000–2022

	**GDP-PPP *per capita* with life expectancy**	**GDP-PPP *per capita* with APC**
**All ASEAN countries (n=230)**	0.674 (0.596, 0.739)****	**-0.382 (-0.488, -0.266)******
**During LIC period (n=51)**	0.663 (0.473, 0.793) ****	0.159 (-0.122, 0.416)
**During L-MIC period (n=95)**	0.506 (0.34, 0.642) ****	0.060 (-0.143, 0.258)
**During U-MIC period (n=38)**	0.216 (-0.112, 0.501)	-0.310 (-0.573, 0.011)
**During HIC period (n=46)**	0.368 (0.087, 0.595)*	0.037 (-0.256, 0.324)
**Cluster 1 (n=69)**	0.638 (0.472, 0.76)****	0.373 (0.149, 0.560)**
**Cluster 2 (n=69)**	0.862 (0.785, 0.912)****	**-0.806 (-0.876, -0.703)******
**Cluster 3 (n=92)**	0.690 (0.565, 0.784)****	**0.641 (0.502, 0.748)******

* p values <0.05
** p values < 0.01
*** p values < 0.001
**** p values < 0.0001

Appendix S5: Results of Generalized Least Squares (GLS) models evaluating the association of Gross Domestic Product *per capita* at purchasing power parity (GDP-PPP *per capita*) with Life Expectancy and with alcohol *per capita* consumption (APC)

Life Expectancy and GDP-PPP *per capita*

10 ASEAN Countries (Life Expectancy with GDP-PPP per capita)

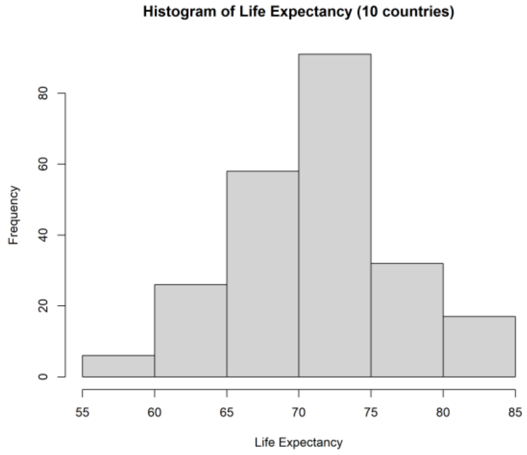

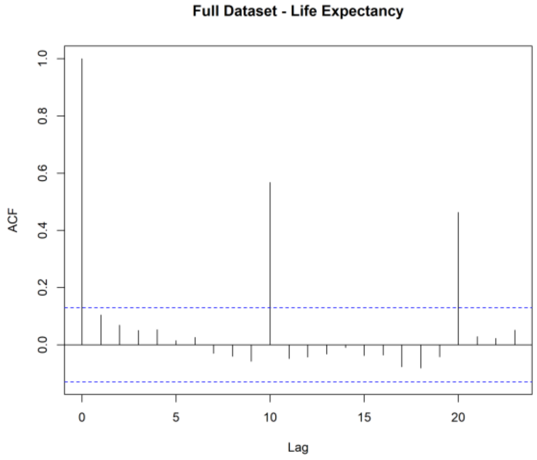


```
## Generalized least squares fit by REML
##   Model: life_expectancy_total ~ gdp_ppp_int_1000
##   Data: data_combined
##        AIC     BIC    logLik
##   1336.002 1349.72 -664.0011
##
## Correlation Structure: Compound symmetry
##  Formula: ~1 | year
##  Parameter estimate(s):
##         Rho
## -0.03164835
##
## Coefficients:
##                      Value Std.Error   t-value p-value
## (Intercept)       68.43412 0.3228588 211.96300       0
## gdp_ppp_int_1000   0.13372 0.0096632  13.83807       0
##
##  Correlation:
##                  (Intr)
## gdp_ppp_int_1000 -0.671
##
## Standardized residuals:
##        Min         Q1        Med         Q3        Max
## -2.8648828 -0.7722125  0.1996953  0.8060654  1.9286175
##
## Residual standard error: 4.291832
## Degrees of freedom: 230 total; 228 residual
```

During low-income country (LIC) periods (Life Expectancy with GDP-PPP per capita)

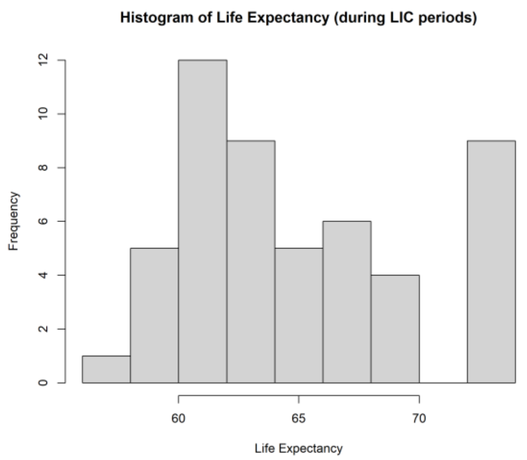

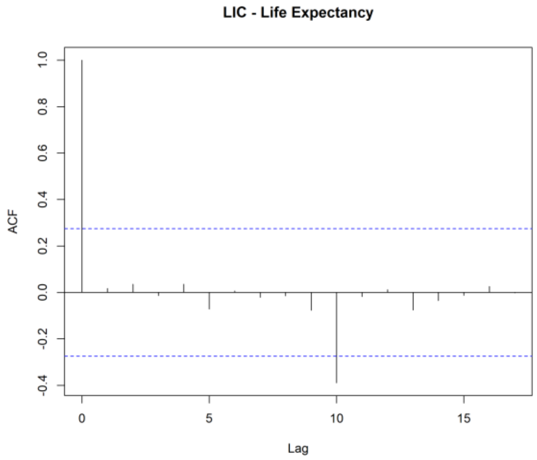


```
## Generalized least squares fit by REML
##   Model: life_expectancy_total ~ gdp_ppp_int_1000
##   Data: data_combined_LIC
##       AIC      BIC    logLik
##   268.437 276.0043 -130.2185
##
## Correlation Structure: Compound symmetry
##  Formula: ~1 | year
##  Parameter estimate(s):
##       Rho
## -0.248521
##
## Coefficients:
##                     Value Std.Error  t-value p-value
## (Intercept)      56.95859 0.7888777 72.20205       0
## gdp_ppp_int_1000  2.83464 0.3249346  8.72374       0
##
##  Correlation:
##                  (Intr)
## gdp_ppp_int_1000 -0.996
##
## Standardized residuals:
##         Min          Q1         Med          Q3         Max
## -2.27585866 -0.88811760  0.02671927  0.47787220  2.23469191
##
## Residual standard error: 3.621671
## Degrees of freedom: 51 total; 49 residual
```

During lower middle-income country periods (L-MIC) (Life Expectancy with GDP-PPP per capita)

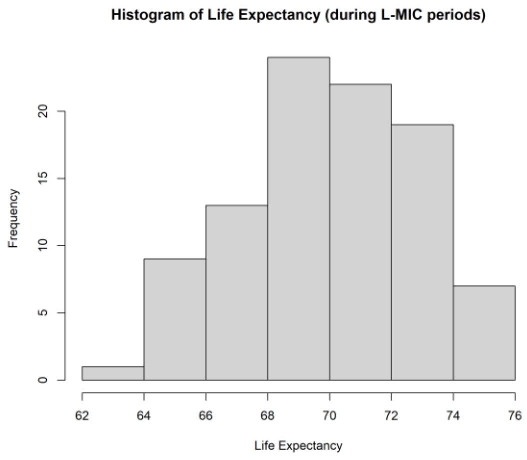

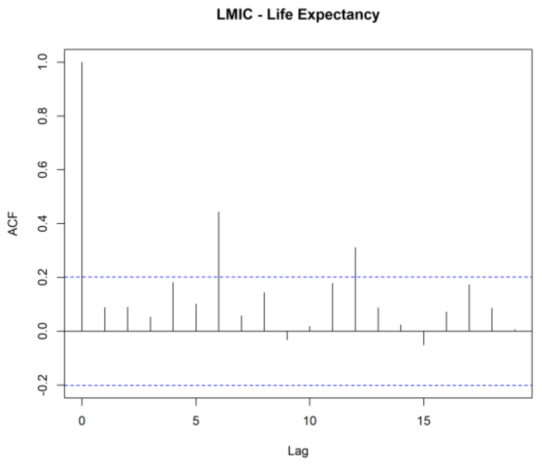


```
## Generalized least squares fit by REML
##   Model: life_expectancy_total ~ gdp_ppp_int_1000
##   Data: data_combined_LMIC
##        AIC      BIC    logLik
##   449.9031 460.0335 -220.9515
##
## Correlation Structure: Compound symmetry
##  Formula: ~1 | year
##  Parameter estimate(s):
##        Rho
## -0.1113973
##
## Coefficients:
##                     Value Std.Error  t-value p-value
## (Intercept)      66.15557 0.7794983 84.86942       0
## gdp_ppp_int_1000  0.52355 0.1019481  5.13544       0
##
##  Correlation:
##                  (Intr)
## gdp_ppp_int_1000 -0.969
##
## Standardized residuals:
##        Min          Q1         Med          Q3         Max
## -2.1173072 -0.8181288  0.4656281  0.7191970  1.8874091
##
## Residual standard error: 2.486421
## Degrees of freedom: 95 total; 93 residual
```

During upper middle income country periods (U-MIC) (Life Expectancy with GDP-PPP per capita)

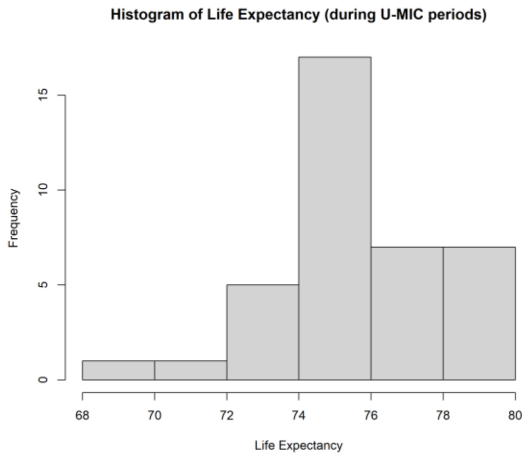

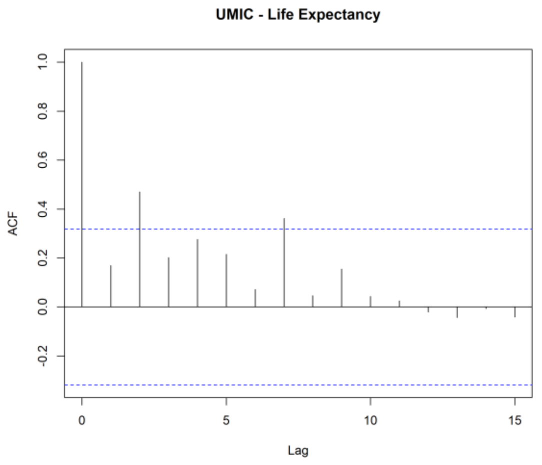


```
## Generalized least squares fit by REML
##   Model: life_expectancy_total ~ gdp_ppp_int_1000
##   Data: data_combined_UMIC
##        AIC      BIC    logLik
##   175.9591 182.2931 -83.97953
##
## Correlation Structure: Compound symmetry
##  Formula: ~1 | year
##  Parameter estimate(s):
##        Rho
## -0.3611836
##
## Coefficients:
##                     Value Std.Error  t-value p-value
## (Intercept)      73.58218  1.372395 53.61591  0.0000
## gdp_ppp_int_1000  0.09596  0.066121  1.45124  0.1554
##
##  Correlation:
##                  (Intr)
## gdp_ppp_int_1000 -0.98
##
## Standardized residuals:
##        Min         Q1        Med         Q3        Max
## -3.0183536 -0.5232446 -0.3350512  0.8904139  1.7857329
##
## Residual standard error: 2.220723
## Degrees of freedom: 38 total; 36 residual
```

During high-income country (HIC) periods (Life Expectancy with GDP-PPP per capita)

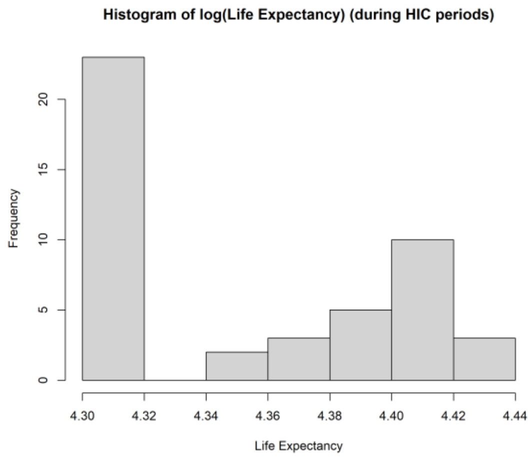

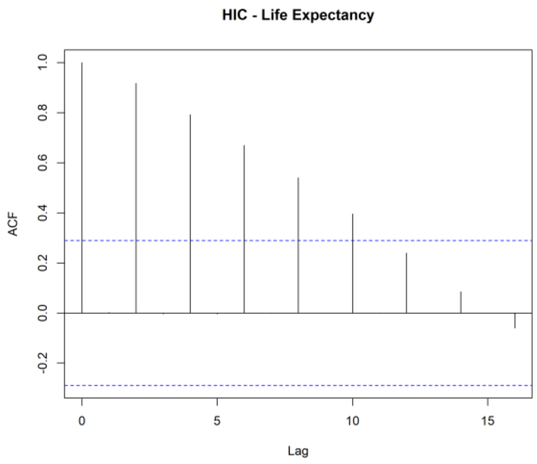


```
## Generalized least squares fit by REML
##   Model: log(life_expectancy_total) ~ gdp_ppp_int_1000
##   Data: data_combined_HIC
##         AIC       BIC   logLik
##   -173.5452 -166.4084 90.77259
##
## Correlation Structure: Compound symmetry
##  Formula: ~1 | year
##  Parameter estimate(s):
##        Rho
## -0.9317189
##
## Coefficients:
##                     Value   Std.Error  t-value p-value
## (Intercept)      4.299711 0.009971657 431.1932       0
## gdp_ppp_int_1000 0.000723 0.000126506   5.7122       0
##
##  Correlation:
##                  (Intr)
## gdp_ppp_int_1000 -0.986
##
## Standardized residuals:
##        Min         Q1        Med         Q3        Max
## -1.1201774 -1.0333199 -0.1345649  1.0669229  1.2489882
##
## Residual standard error: 0.04248065
## Degrees of freedom: 46 total; 44 residual
```

Grouping by clusters

Cluster 1 (Life Expectancy with GDP-PPP per capita)

Brunei Darussalam, Indonesia, Malaysia

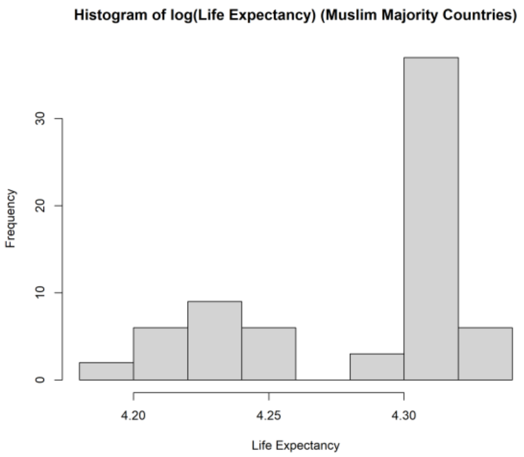

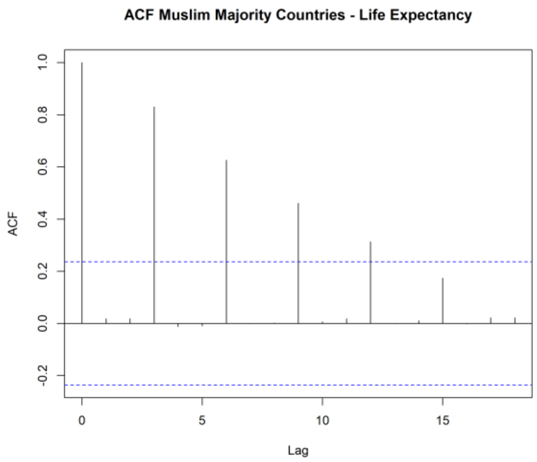


```
## Generalized least squares fit by REML
##   Model: log(life_expectancy_total) ~ gdp_ppp_int_1000
##   Data: cluster1
##         AIC       BIC   logLik
##   -259.1571 -250.3383 133.5785
##
## Correlation Structure: Compound symmetry
##  Formula: ~1 | year
##  Parameter estimate(s):
##        Rho
## -0.3840114
##
## Coefficients:
##                     Value   Std.Error  t-value p-value
## (Intercept)      4.250871 0.005814075 731.1345       0
## gdp_ppp_int_1000 0.000931 0.000154290   6.0372       0
##
##  Correlation:
##                  (Intr)
## gdp_ppp_int_1000 -0.943
##
## Standardized residuals:
##        Min          Q1         Med          Q3         Max
## -2.0991584 -0.5505858 -0.3300065  1.1180965  1.5956793
##
## Residual standard error: 0.03349936
## Degrees of freedom: 69 total; 67 residual
```

Cluster 2 (Life Expectancy with GDP-PPP per capita)

Philippines, Singapore, Thailand

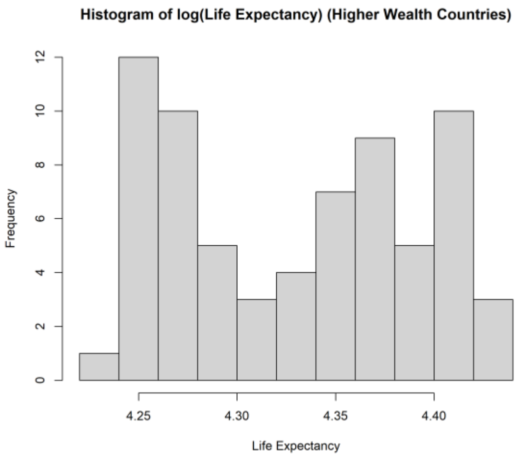

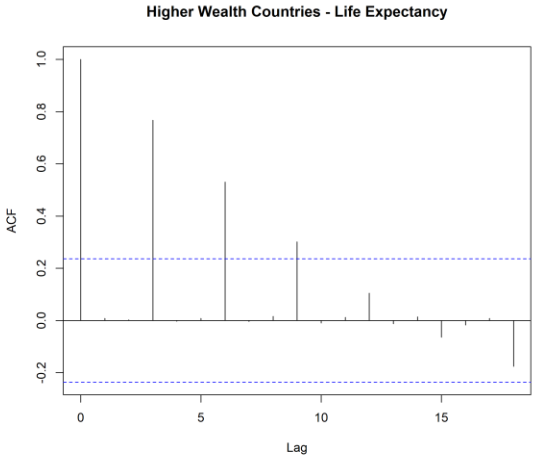


```
## Generalized least squares fit by REML
##   Model: log(life_expectancy_total) ~ gdp_ppp_int_1000
##   Data: cluster2
##         AIC       BIC   logLik
##   -261.2973 -252.4785 134.6486
##
## Correlation Structure: Compound symmetry
##  Formula: ~1 | year
##  Parameter estimate(s):
##        Rho
## -0.3781369
##
## Coefficients:
##                     Value   Std.Error   t-value p-value
## (Intercept)      4.279403 0.004050324 1056.5581       0
## gdp_ppp_int_1000 0.001531 0.000107528   14.2406       0
##
##  Correlation:
##                  (Intr)
## gdp_ppp_int_1000 -0.878
##
## Standardized residuals:
##          Min           Q1          Med           Q3          Max
## -2.428314989 -0.762275240  0.005432712  0.510435819  1.982078472
##
## Residual standard error: 0.03258742
## Degrees of freedom: 69 total; 67 residual
```

Cluster 3 (Life Expectancy with GDP-PPP per capita)

Cambodia, Lao People's Democratic Republic, Myanmar, Viet Nam

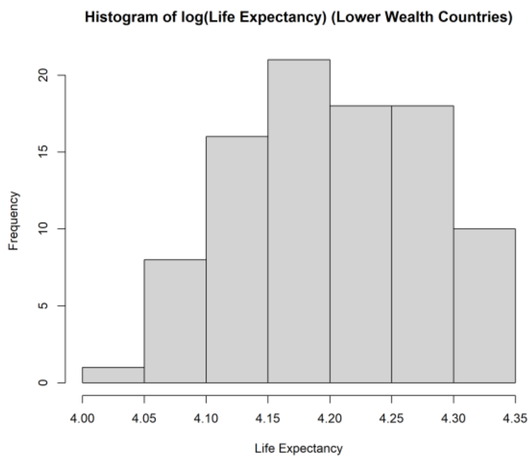

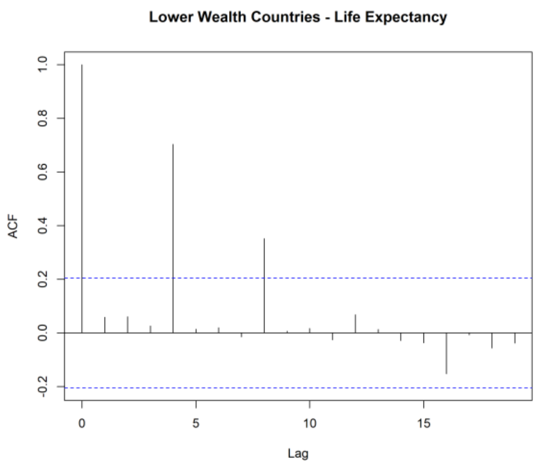


```
## Generalized least squares fit by REML
##   Model: log(life_expectancy_total) ~ gdp_ppp_int_1000
##   Data: cluster3
##         AIC       BIC   logLik
##   -265.4271 -255.4278 136.7135
##
## Correlation Structure: Compound symmetry
##  Formula: ~1 | year
##  Parameter estimate(s):
##        Rho
## -0.2448372
##
## Coefficients:
##                     Value   Std.Error  t-value p-value
## (Intercept)      4.124356 0.006462773 638.1713       0
## gdp_ppp_int_1000 0.017047 0.001253661  13.5976       0
##
##  Correlation:
##                  (Intr)
## gdp_ppp_int_1000 -0.895
##
## Standardized residuals:
##        Min         Q1        Med         Q3        Max
## -2.5544905 -0.6622787 -0.4049226  0.7712184  2.1305642
##
## Residual standard error: 0.05357441
## Degrees of freedom: 92 total; 90 residual
```

APC with GDP-PPP *per capita*

10 ASEAN Countries (APC with GDP-PPP per capita)

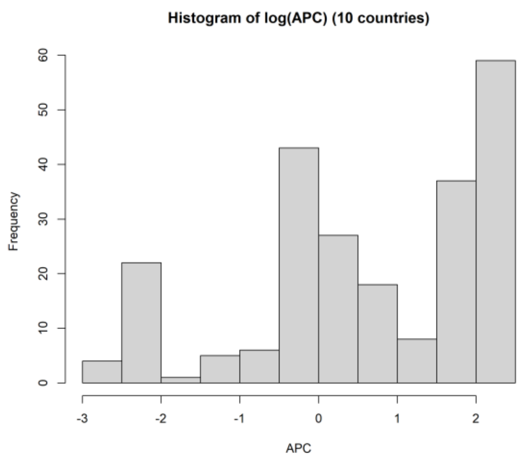

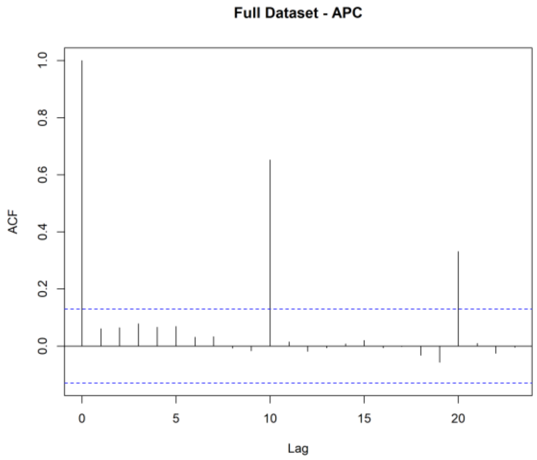


```
## Generalized least squares fit by REML
##   Model: log(total_apc) ~ gdp_ppp_int_1000
##   Data: data_combined
##        AIC      BIC    logLik
##   833.2029 846.9203 -412.6014
##
## Correlation Structure: Compound symmetry
##  Formula: ~1 | year
##  Parameter estimate(s):
##         Rho
## -0.06640385
##
## Coefficients:
##                       Value  Std.Error   t-value p-value
## (Intercept)       0.8806649 0.09470368  9.299163   0e+00
## gdp_ppp_int_1000 -0.0112760 0.00325405 -3.465216   6e-04
##
##  Correlation:
##                  (Intr)
## gdp_ppp_int_1000 -0.771
##
## Standardized residuals:
##        Min         Q1        Med         Q3        Max
## -2.4163068 -0.6084105  0.2937690  0.8964810  1.1929545
##
## Residual standard error: 1.443106
## Degrees of freedom: 230 total; 228 residual
```

During low-income country (LIC) periods (APC with GDP-PPP per capita)

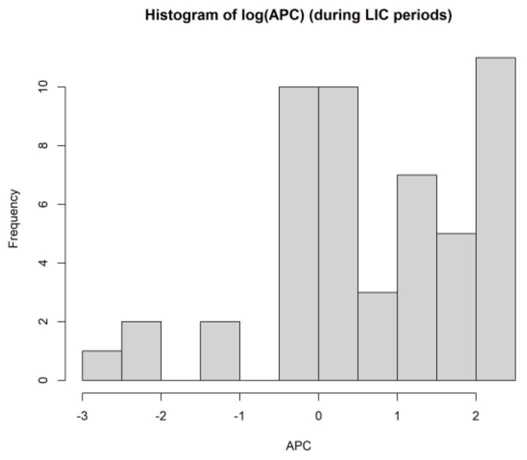

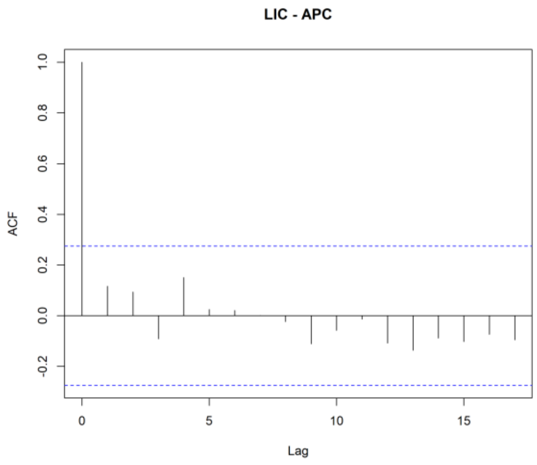


```
## Generalized least squares fit by REML
##   Model: log(total_apc) ~ gdp_ppp_int_1000
##   Data: data_combined_LIC
##        AIC      BIC    logLik
##   179.1639 186.7312 -85.58195
##
## Correlation Structure: Compound symmetry
##  Formula: ~1 | year
##  Parameter estimate(s):
##         Rho
## -0.09640037
##
## Coefficients:
##                      Value Std.Error   t-value p-value
## (Intercept)      0.2875401 0.4838465 0.5942797  0.5551
## gdp_ppp_int_1000 0.1266238 0.1662358 0.7617123  0.4499
##
##  Correlation:
##                  (Intr)
## gdp_ppp_int_1000 -0.95
##
## Standardized residuals:
##         Min          Q1         Med          Q3         Max
## -2.74310349 -0.51135243 -0.08692726  0.78402160  1.27645942
##
## Residual standard error: 1.284823
## Degrees of freedom: 51 total; 49 residual
```

During lower middle-income country periods (L-MIC) (APC with GDP-PPP per capita)

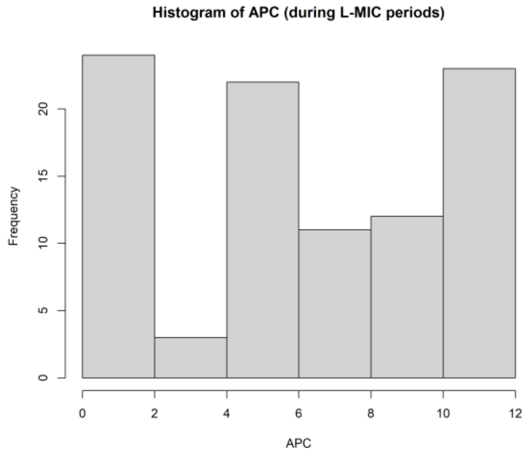

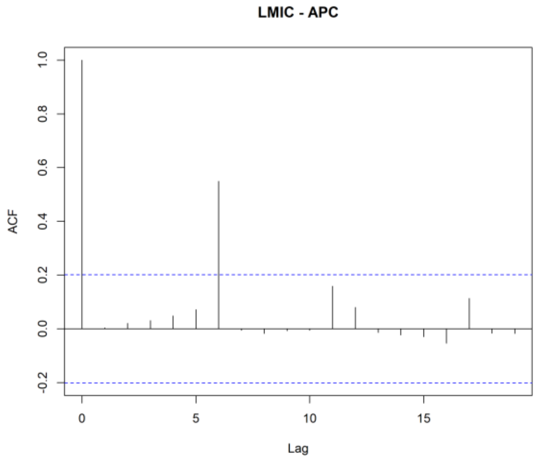


```
## Generalized least squares fit by REML
##   Model: total_apc ~ gdp_ppp_int_1000
##   Data: data_combined_LMIC
##        AIC      BIC    logLik
##   517.7967 527.9271 -254.8983
##
## Correlation Structure: Compound symmetry
##  Formula: ~1 | year
##  Parameter estimate(s):
##       Rho
## -0.195699
##
## Coefficients:
##                    Value Std.Error  t-value p-value
## (Intercept)      5.52367 0.7356365 7.508695  0.0000
## gdp_ppp_int_1000 0.03561 0.0947308 0.375908  0.7078
##
##  Correlation:
##                  (Intr)
## gdp_ppp_int_1000 -0.992
##
## Standardized residuals:
##         Min          Q1         Med          Q3         Max
## -1.50651982 -0.95324640  0.03228035  1.03654598  1.45310819
##
## Residual standard error: 3.912266
## Degrees of freedom: 95 total; 93 residual
```

During upper middle-income country periods (U-MIC) (APC with GDP-PPP per capita)

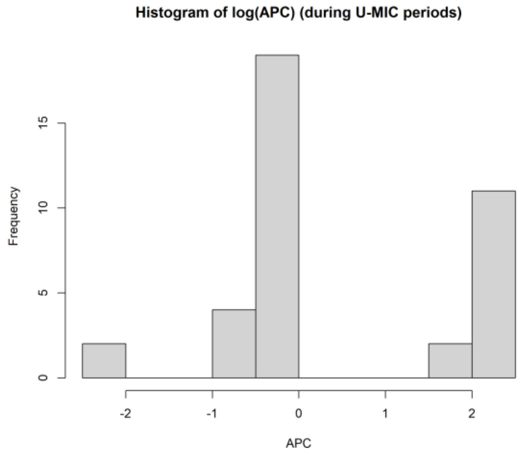

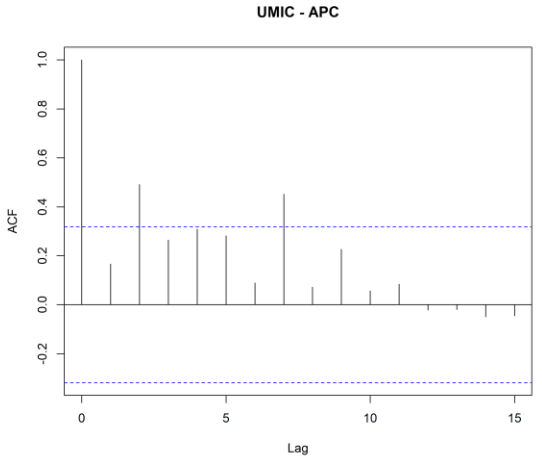


```
## Generalized least squares fit by REML
##   Model: log(total_apc) ~ gdp_ppp_int_1000
##   Data: data_combined_UMIC
##        AIC      BIC    logLik
##   134.7191 141.0532 -63.35954
##
## Correlation Structure: Compound symmetry
##  Formula: ~1 | year
##  Parameter estimate(s):
##        Rho
## -0.3893296
##
## Coefficients:
##                       Value Std.Error    t-value p-value
## (Intercept)       0.9312022 0.7736314  1.2036770  0.2366
## gdp_ppp_int_1000 -0.0293472 0.0371056 -0.7909092  0.4342
##
##  Correlation:
##                  (Intr)
## gdp_ppp_int_1000 -0.982
##
## Standardized residuals:
##        Min         Q1        Med         Q3        Max
## -2.2542292 -0.7043295 -0.3034289  1.2682309  1.4135999
##
## Residual standard error: 1.261396
## Degrees of freedom: 38 total; 36 residual
```

During high-income country (HIC) periods (APC with GDP-PPP per capita)

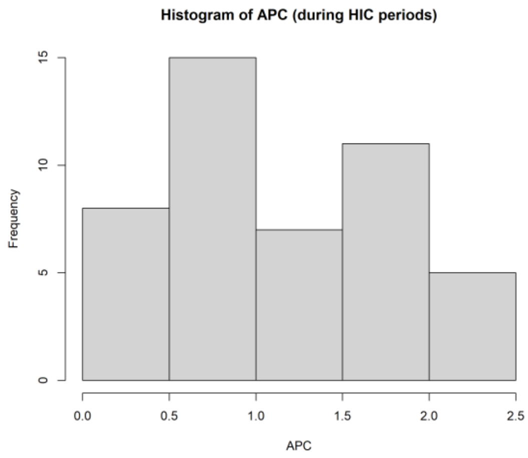

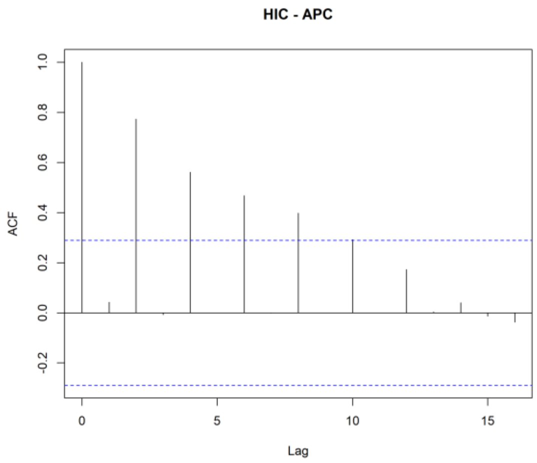


```
## Generalized least squares fit by REML
##   Model: total_apc ~ gdp_ppp_int_1000
##   Data: data_combined_HIC
##       AIC      BIC    logLik
##   74.9304 82.06716 -33.4652
##
## Correlation Structure: Compound symmetry
##  Formula: ~1 | year
##  Parameter estimate(s):
##        Rho
## -0.8814548
##
## Coefficients:
##                      Value  Std.Error  t-value p-value
## (Intercept)      0.7170564 0.19200592 3.734554  0.0005
## gdp_ppp_int_1000 0.0051101 0.00243477 2.098795  0.0416
##
##  Correlation:
##                  (Intr)
## gdp_ppp_int_1000 -0.986
##
## Standardized residuals:
##         Min          Q1         Med          Q3         Max
## -1.64364288 -0.67459052 -0.02487705  0.78728981  2.14778776
##
## Residual standard error: 0.6311186
## Degrees of freedom: 46 total; 44 residual
```

Grouping by clusters

Cluster 1 (APC with GDP-PPP per capita)

Brunei Darussalam, Indonesia, Malaysia

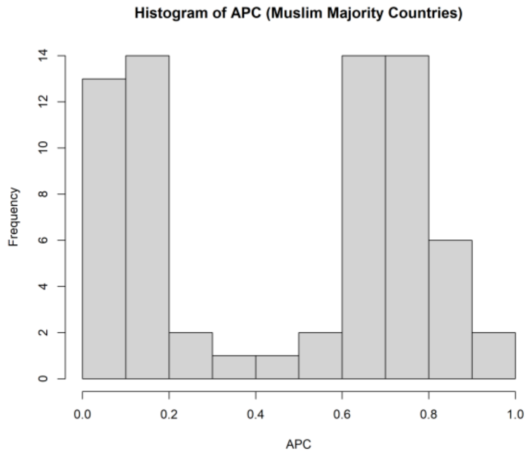

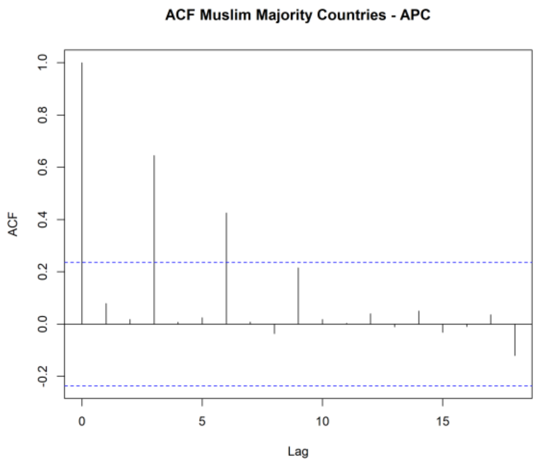


```
## Generalized least squares fit by REML
##   Model: total_apc ~ gdp_ppp_int_1000
##   Data: cluster1
##        AIC      BIC    logLik
##   37.78014 46.59891 -14.89007
##
## Correlation Structure: Compound symmetry
##  Formula: ~1 | year
##  Parameter estimate(s):
##        Rho
## -0.2951904
##
## Coefficients:
##                      Value  Std.Error  t-value p-value
## (Intercept)      0.3039591 0.05103689 5.955674  0.0000
## gdp_ppp_int_1000 0.0040720 0.00129591 3.142193  0.0025
##
##  Correlation:
##                  (Intr)
## gdp_ppp_int_1000 -0.902
##
## Standardized residuals:
##        Min         Q1        Med         Q3        Max
## -1.8895773 -0.8380579  0.2492693  0.8967968  1.6825293
##
## Residual standard error: 0.2861859
## Degrees of freedom: 69 total; 67 residual
```

Cluster 2 (APC with GDP-PPP per capita)

Philippines, Singapore, Thailand

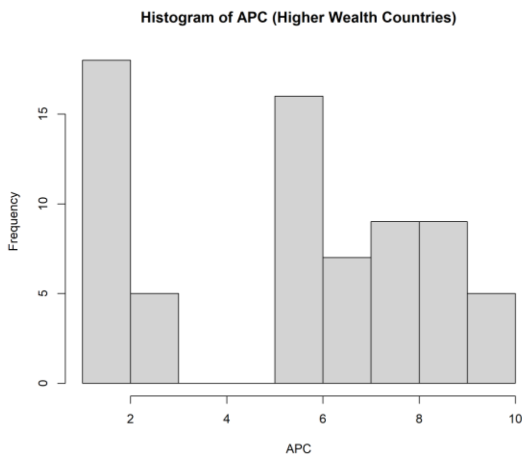

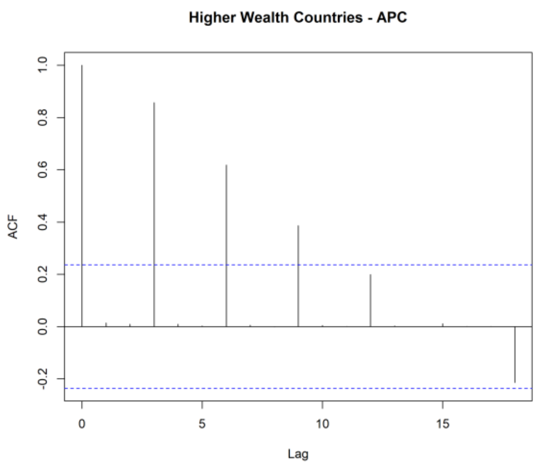


```
## Generalized least squares fit by REML
##   Model: total_apc ~ gdp_ppp_int_1000
##   Data: cluster2
##        AIC      BIC    logLik
##   260.3251 269.1439 -126.1626
##
## Correlation Structure: Compound symmetry
##  Formula: ~1 | year
##  Parameter estimate(s):
##        Rho
## -0.4858383
##
## Coefficients:
##                      Value  Std.Error  t-value p-value
## (Intercept)       5.970305 0.13381394 44.61647       0
## gdp_ppp_int_1000 -0.021195 0.00381466 -5.55628       0
##
##  Correlation:
##                  (Intr)
## gdp_ppp_int_1000 -0.943
##
## Standardized residuals:
##         Min          Q1         Med          Q3         Max
## -1.56659660 -1.14825882 -0.02235026  0.99306123  1.71214845
##
## Residual standard error: 2.196439
## Degrees of freedom: 69 total; 67 residual
```

Cluster 3 (APC with GDP-PPP per capita)

Cambodia, Lao People's Democratic Republic, Myanmar, Viet Nam

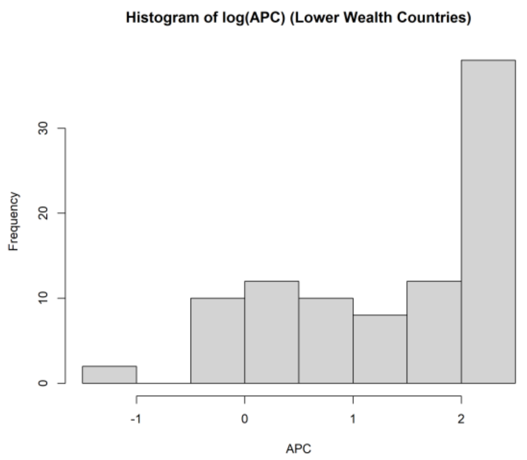

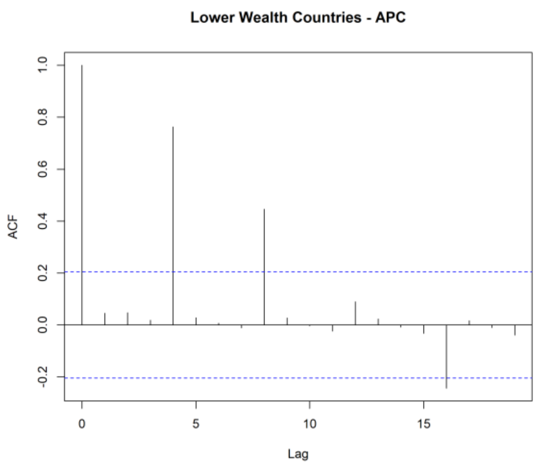


```
## Generalized least squares fit by REML
##   Model: log(total_apc) ~ gdp_ppp_int_1000
##   Data: cluster3
##         AIC    BIC    logLik
##   214.2508 224.25 -103.1254
##
## Correlation Structure: Compound symmetry
##  Formula: ~1 | year
##  Parameter estimate(s):
##         Rho
## -0.2786578
##
## Coefficients:
##                      Value  Std.Error   t-value p-value
## (Intercept)      0.4829744 0.07749634  6.232223       0
## gdp_ppp_int_1000 0.1888397 0.01508402 12.519189       0
##
##  Correlation:
##                  (Intr)
## gdp_ppp_int_1000 -0.898
##
## Standardized residuals:
##         Min          Q1         Med          Q3         Max
## -2.47620745 -0.93091971  0.08840355  0.72229071  1.65388594
##
## Residual standard error: 0.8059694
## Degrees of freedom: 92 total; 90 residual
```

References

1 World Health Organization. Alcohol, total per capita (15+ years) consumption (in litres of pure alcohol). Geneva, Switzerland: WHO; 2025 [22/03/2025]; Available from: https://www.who.int/data/gho/indicator-metadata-registry/imr-details/465.
2 Association of Southeast Asian Nations (ASEAN). Home page. Jakarta: The ASEAN Secretariat; 2025 [17/03/2025]; Available from: https://asean.org/.
3 World Bank. World Bank country classifications by income level for 2024-2025. The World Bank Group; 2024 [22/03/2025]; Available from: https://blogs.worldbank.org/en/opendata/world-bank-country-classifications-by-income-level-for-2024-2025
4 World Health Organization. Gross Domestic Product (GDP), per capita, international $ (PPP-adjusted). Geneva, Switzerland: WHO; 2025.
5 World Health Organization. The SAFER Initiative: A world free from alcohol related harm. Geneva: WHO; 2023 [25/07/2024]; Available from: https://www.who.int/initiatives/SAFER.
6 World Bank. GDP per capita, PPP (current international $). 2024 [10/03/2024]; Available from: https://data.worldbank.org/indicator/NY.GDP.PCAP.PP.CD.
7 World Bank. Life expectancy at birth, total (years). New York: The World Bank; 2024 [17/03/2025]; Available from: https://data.worldbank.org/indicator/SP.DYN.LE00.IN.
8 World Health Organization. World Health Statistics. Geneva, Switzerland: WHO; 2025.
9 Poznyak V, Fleischmann A, Rekve D, Rylett M, Rehm J, Gmel G. The World Health Organization's global monitoring system on alcohol and health. Alcohol Res. 2013; 35:244-9. doi:
10 Rehm J, Klotsche J, Patra J. Comparative quantification of alcohol exposure as risk factor for global burden of disease. Int J Methods Psychiatr Res. 2007; 16:66-76. doi: 10.1002/mpr.204.
11 Probst C, Manthey J, Merey A, Rylett M, Rehm J. Unrecorded alcohol use: a global modelling study based on nominal group assessments and survey data. Addiction. 2018; 113:1231-41. doi: 10.1111/add.14173.
12 Probst C, Fleischmann A, Gmel G, Poznyak V, Rekve D, Riley L, et al. The global proportion and volume of unrecorded alcohol in 2015. J Glob Health. 2019; 9:010421. doi: 10.7189/jogh.09.010421.
13 World Health Organization. STEPwise approach to NCD risk factor surveillance (STEPS). Geneva: WHO; 2025 [17/04/2025]; Available from: https://www.who.int/teams/noncommunicable-diseases/surveillance/systems-tools/steps.
14 UN Tourism. Tourism Statistics Database. Madrid, Spain: UN Tourism; 2025 [29/12/2025]; Available from: https://www.untourism.int/tourism-statistics/tourism-statistics-database.
15 World Population Review. Muslim Population by Country 2025. Lancaster, PA: World Population Review; 2025 [20/03/2025]; Available from: https://worldpopulationreview.com/country-rankings/muslim-population-by-country.
16 Assanangkornchai S, Wichaidit W, Amul GGH, Ang IYH, Chaiyasong S, Chhoun P, et al. Alcohol control policy in Southeast Asia: a descriptive review. Asia-Pacific Journal of Public Health. 2026 (in press). doi: 10.1177/10105395251414918.
17 Rekve D, Banatvala N, Karpati A, Tarlton D, Westerman L, Sperkova K, et al. Prioritising action on alcohol for health and development. BMJ. 2019; 367:l6162. doi: 10.1136/bmj.l6162.
18 World Health Organization. Global report on the use of alcohol taxes, 2023. Geneva: World Health Organization; 2023 [20/03/2025]; Available from: https://www.who.int/publications/i/item/9789240086104.
19 Brand DA, Saisana M, Rynn LA, Pennoni F, Lowenfels AB. Comparative analysis of alcohol control policies in 30 countries. PLoS Med. 2007; 4:e151. doi: 10.1371/journal.pmed.0040151.
20 Casswell S, Huckle T, Parker K, Romeo J, Graydon-Guy T, Leung J, et al. Benchmarking alcohol policy based on stringency and impact: The International Alcohol Control (IAC) policy index. PLOS Glob Public Health. 2022; 2:e0000109. doi: 10.1371/journal.pgph.0000109.

21 Madureira-Lima J, Galea S. Alcohol control policies and alcohol consumption: an international comparison of 167 countries. J Epidemiol Community Health. 2018; 72:54-60. doi: 10.1136/jech-2017-209350.
22 Naimi T, Stockwell T, Giesbrecht N, Wettlaufer A, Vallance K, Farrell-Low A, et al. Canadian Alcohol Policy Evaluation (CAPE) 3.0: Policy Scoring Rubric (Federal). Victoria, BC: Canadian institute for Substance Use Research, University of Victoria; 2023.
23 Naimi TS, Blanchette J, Nelson TF, Nguyen T, Oussayef N, Heeren TC, et al. A new scale of the U.S. alcohol policy environment and its relationship to binge drinking. Am J Prev Med. 2014; 46:10-6. doi: 10.1016/j.amepre.2013.07.015.
24 Pan American Health Organization. Alcohol Policy Scoring: Assessing the level of implementation of the WHO Global strategy to reduce the harmful use of alcohol in the Region of the Americas. Washington, D.C.: PAHO; 2018.
25 Månsson A, van der Velde L, Karlsson T, Beekmann L, Jonsson Stenberg E, Haagsma J, et al. Alcohol control policy and alcohol-attributable disease burden in Finland and the Baltic countries: A longitudinal study 1995-2019. Drug Alcohol Rev. 2024; 43:1338-48. doi: 10.1111/dar.13901.
26 Amul GGH, Etter JF. Comparing Tobacco and Alcohol Policies From a Health Systems Perspective: The Cases of the Philippines and Singapore. Int J Public Health. 2022; 67:1605050. doi: 10.3389/ijph.2022.1605050.
27 World Health Organization. Global strategy to reduce the harmful use of alcohol. Geneva, Switzerland: World Health Organization; 2010 [22/05/2025]; Available from: https://www.who.int/publications/i/item/9789241599931.
28 Rehm J, Badaras R, Ferreira-Borges C, Galkus L, Gostautaite Midttun N, Gobiņa I, et al. Impact of the WHO "best buys" for alcohol policy on consumption and health in the Baltic countries and Poland 2000-2020. Lancet Reg Health Eur. 2023; 33:100704. doi: 10.1016/j.lanepe.2023.100704.
29 Chisholm D, Moro D, Bertram M, Pretorius C, Gmel G, Shield K, et al. Are the "Best Buys" for Alcohol Control Still Valid? An Update on the Comparative Cost-Effectiveness of Alcohol Control Strategies at the Global Level. Journal of studies on alcohol and drugs. 2018; 79:514-22. doi: 10.15288/jsad.2018.79.514.
30 World Health Organization. Tackling NCDs: best buys and other recommended interventions for the prevention and control of noncommunicable diseases, 2nd ed. Geneva, Switzerland: World Health Organization; 2024 [22/05/2025]; Available from: https://www.who.int/publications/i/item/9789240091078.
31 Lachenmeier DW, Neufeld M, Rehm J. The Impact of Unrecorded Alcohol Use on Health: What Do We Know in 2020? Journal of studies on alcohol and drugs. 2021; 82:28-41. doi: 10.15288/jsad.2021.82.28.
32 Rehm J, Kailasapillai S, Larsen E, Rehm MX, Samokhvalov AV, Shield KD, et al. A systematic review of the epidemiology of unrecorded alcohol consumption and the chemical composition of unrecorded alcohol. Addiction. 2014; 109:880-93. doi: 10.1111/add.12498.
33 World Health Organization. Global prices and taxes on alcoholic beverages. 2024 [31/12/2025]. Available from: https://www.who.int/data/gho/data/themes/topics/taxes-on-alcoholic-beverages
